# Transcriptomic signatures reveal systemic adaptations and immune modulation in response to training and competitive racing in horses

**DOI:** 10.1002/evj.70154

**Published:** 2026-03-07

**Authors:** Izabela Dąbrowska, Jowita Grzędzicka‐Agko, Paula Kiełbik, Michał Trela, Olga Witkowska‐Piłaszewicz

**Affiliations:** ^1^ Department of Large Animal Diseases and Clinic, Institute of Veterinary Medicine Warsaw University of Life Sciences Warsaw Poland

**Keywords:** gene expression profiling, horse, immune response, overtraining biomarkers, race‐induced stress, training adaptation, transcriptomics

## Abstract

**Background:**

The molecular mechanisms underlying adaptation to physical exertion and racing stress in horses remain incompletely understood. Peripheral blood transcriptomics offers a minimally invasive method to monitor systemic responses to exercise and identify biomarkers of adaptation or overload.

**Objectives:**

To evaluate transcriptomic changes in peripheral blood of racehorses during different phases of training and competition and to identify molecular markers of physiological adaptation and race‐induced stress.

**Study Design:**

Prospective transcriptomic profiling of trained racehorses across three exercise conditions.

**Methods:**

Forty racehorses (29 Arabian, 11 Thoroughbred) were sampled before (p0) and after (p1) exercise at three stages: initial training (T1), mid‐season training (T2) and racing (R). RNA‐seq was performed, followed by differential expression analysis (DESeq2) and pathway enrichment (g:Profiler, DAVID). Identified differentially expressed genes were integrated into STRING protein–protein interaction condition‐exclusive subnetworks (Merge tool in Cytoscape) to compare the transcriptomes between conditions.

**Results:**

Distinct molecular programmes were identified at each stage. At T1, immediate‐early response genes such as *FOS*, *FOSB* and *HSPA6* were strongly up‐regulated, reflecting acute stress and immune activation. At T2, immune‐related transcripts (*KLRD1*, *CCL4*, *PRF1*) remained enriched, but genes linked to remodelling and adaptation, including *DNAJA1*, *HSPH1* and *CXCR4*, became prominent, suggesting a shift toward recovery and regulatory processes. Post‐race, chemokines such as CCL5 and stress markers (*HSP90* family, *JUN*) were highly induced, accompanied by widespread transcriptomic divergence and down‐regulation of certain immune regulators (*IL18*, *ARHGAP44*), indicating both heightened innate activation and transient immune suppression.

**Main Limitations:**

Transcriptomic profiling was limited to peripheral blood, which may not reflect tissue‐specific responses. Only three sampling points were included, potentially overlooking transient transcriptomic changes.

**Conclusions:**

Transcriptomic dynamics in blood reflect the transition from early immune activation (T1), through adaptation (T2), to stress‐related activation post‐race (R). This approach offers promising molecular biomarkers for monitoring training adaptation and detecting physiological overload in equine athletes.

## Zusammenfassung

**Hintergrund:**

Die molekularen Mechanismen, die der Anpassung an körperliche Anstrengung und Rennstress bei Pferden zugrunde liegen, sind noch nicht vollständig geklärt. Die Transkriptomik des peripheren Blutes bietet eine minimalinvasive Methode zur Überwachung systemischer Reaktionen auf körperliche Belastung und zur Identifizierung von Biomarkern für Anpassung oder Überlastung.

**Ziele:**

Bewertung der transkriptomischen Veränderungen im peripheren Blut von Rennpferden während verschiedener Trainings‐ und Wettkampfphasen und Identifizierung molekularer Marker für physiologische Anpassung und rennbedingten Stress.

**Studiendesign:**

Prospektive Transkriptom‐Profilierung von trainierten Rennpferden unter drei Trainingsbedingungen.

**Methoden:**

Vierzig Rennpferde (29 Araber, 11 Vollblüter) wurden vor (p0) und nach (p1) dem Training in drei Phasen untersucht: zu Beginn des Trainings (T1), in der Mitte der Saison (T2) und während des Rennens (R). Es wurde eine RNA‐Sequenzierung durchgeführt, gefolgt von einer Differentialexpressionsanalyse (DESeq2) und einer Signalweg‐Anreicherung (g:Profiler, DAVID). Identifizierte DEGs wurden in STRING PPIs bedingungsspezifische Subnetzwerke integriert (Merge‐Tool in Cytoscape), um die Transkriptome zwischen den Bedingungen zu vergleichen.

**Ergebnisse:**

In jeder Phase wurden unterschiedliche molekulare Programme identifiziert. Bei T1 waren sofortige Frühreaktionsgene wie FOS, FOSB und HSPA6 stark hochreguliert, was auf akuten Stress und eine Immunaktivierung hindeutet. Bei T2 blieben immunbezogene Transkripte (KLRD1, CCL4, PRF1) angereichert, aber Gene, die mit Umbau und Anpassung in Verbindung stehen, darunter DNAJA1, HSPH1 und CXCR4, traten in den Vordergrund, was auf eine Verlagerung hin zu Erholungs‐ und Regulationsprozessen hindeutet. Nach dem Rennen waren Chemokine wie CCL5 und Stressmarker (HSP90‐Familie, JUN) stark induziert, begleitet von einer weitreichenden transkriptomischen Divergenz und einer Herunterregulierung bestimmter Immunregulatoren (IL18, ARHGAP44), was sowohl auf eine verstärkte angeborene Aktivierung als auch auf eine vorübergehende Immunsuppression hindeutet.

**Hauptlimitationen:**

Die Transkriptom‐Profilierung beschränkte sich auf peripheres Blut, was möglicherweise nicht die gewebespezifischen Reaktionen widerspiegelt. Es wurden nur drei Probenentnahmepunkte berücksichtigt, wodurch möglicherweise vorübergehende transkriptomische Veränderungen übersehen wurden.

**Schlußfolgerungen:**

Die Transkriptomdynamik im Blut spiegelt den Übergang von der frühen Immunaktivierung (T1) über die Anpassung (T2) bis hin zur stressbedingten Aktivierung nach dem Rennen (R) wider. Dieser Ansatz bietet vielversprechende molekulare Biomarker für die Überwachung der Trainingsanpassung und die Erkennung physiologischer Überlastung bei Sportpferden.

## 1 INTRODUCTION

Physical activity significantly influences the physiology of athletic animals, and horses are a well‐established model for studying exercise‐induced adaptations in the musculoskeletal, cardiovascular and immune systems.^1^, ^2^ In equine athletes, both acute high‐intensity effort (racing) and chronic training stimulus (conditioning) trigger broad cellular and molecular responses. While training aims to improve aerobic capacity,^3^ muscle endurance,^4^ and immune tolerance,^5^ competitive racing imposes an acute stress challenge often associated with transient inflammatory and metabolic disturbances.^6^, ^7^, ^8^ Although controlled exercise enhances oxygen delivery^9^ and metabolic efficiency, inadequate recovery may lead to chronic fatigue and performance decline, known as overtraining syndrome.^10^ A key challenge in equine performance medicine is therefore the early discrimination between adaptive responses and early signs of physiological overload.

Transcriptomic profiling offers a promising tool to explore these transitions, providing a systemic, minimally invasive snapshot of exercise‐induced molecular changes.^11^, ^12^ In Thoroughbreds, exercise modulates transcripts related to mitochondrial function (*MRPS21*, *SLC25A29*), oxidative metabolism (*PGC‐1α*, *HIF‐1α*) and down‐regulates muscle growth inhibitors like *MSTN*.^13^, ^14^, ^15^ In Arabians, trained for endurance, transcriptomic adaptations include aerobic and lipid metabolism (*PPARA*, *PPARD*, *PLIN2*), vascular remodelling (*VEGFB*, *VEGFD*) and antioxidant regulation.^12^, ^16^, ^17^, ^18^ Additional studies highlight stress‐ and immune‐related transcriptional activation during prolonged exertion, including increased *TLR4*, *CXCL8*, *IL22A2* and *HSPA1A* expression.^19^, ^20^, ^21^

Despite these advances, most data are derived from isolated time points or rest‐state comparisons. Little is known about the dynamic progression of transcriptomic responses throughout a real‐life training and racing cycle. This study aims to characterise longitudinal transcriptomic responses to exercise in peripheral blood of racehorses sampled at three key time points: the start of the training season (T1), mid‐season training (T2) and post‐race (R). We hypothesised that these stages reflect a molecular trajectory from early immune activation, through adaptive stabilisation, to stress‐related divergence. Our objective is to identify gene signatures and pathways that differentiate adaptation from overload, contributing to the development of blood‐based biomarkers for monitoring fitness, fatigue and welfare in equine athletes.

## 2 MATERIALS AND METHODS

### 2.1 Animals

The study included 40 racehorses: 29 Arabian (9 mares and 20 stallions) and 11 Thoroughbred (5 mares and 6 stallions), aged between 2 and 7 years (median: 3 years), all trained by the same professional trainer. The horses followed a consistent, high‐intensity race training regimen. Each horse was stabled individually with suitable bedding, had daily outdoor turnout depending on weather conditions and received a uniform diet consisting of high‐quality hay, commercial concentrate feed and vitamin‐mineral supplements, with constant access to clean water. The sample size was determined based on previous transcriptomic studies in equine athletes, which report robust gene expression changes (log_2_FC > 1.5, FDR < 0.05) with group sizes ranging from 8 to 12 per condition.^16^, ^20^ Assuming an effect size (Cohen's *d*) of 1.0 for key transcriptomic parameters (e.g., expression of immune‐ or stress‐related genes), a power analysis using G*Power (v3.1) indicated that a sample size of at least 16 animals per group (pre vs. post) would be required to achieve 80% power at alpha = 0.05 (two‐tailed paired design). Our final sample (*n* = 32–39 across time points) exceeded this minimum, ensuring sufficient statistical power for longitudinal comparisons.

Training sessions were conducted 6 days a week, weather permitting and were structured as follows: a warm‐up phase involving 10 min of walking under saddle, followed by 800 m of trotting and 800 m of relaxed cantering. The conditioning phase consisted of 1400 to 2400 m of moderate‐paced cantering (16–22 s per 200 m). This was followed by a high‐intensity gallop phase over distances of 500–1200 meters at 80–100% of maximal speed (13–15 s per 200 m). Each session concluded with a 30‐ to 40‐min cool‐down on a mechanical walker. Gallop‐based high‐intensity sessions were scheduled every 5–10 days to optimise performance. Based on clinical and biochemical evaluations, all horses met the inclusion criteria and were deemed suitable for the transcriptomic analysis.

### 2.2 Sample collection

Blood sampling was carried out to assess both baseline values and the effects of exercise on routine haematological parameters during standard veterinary procedures as part of ongoing health monitoring and athletic performance assessment. Sampling was performed at three phases of the training programme: the start of the season (T1, initial training; *n* = 35), after 6–10 weeks of continued training (T2, mid‐training; *n* = 39), during high‐intensity sessions comprising training gallops and during official flat races (R, race phase; pre‐ and post‐effort; *n* = 32). At T1 and T2, sampling days were selected to coincide with gallop‐based high‐intensity sessions designed to match acute workload between stages. These sessions followed an identical structure: warm‐up (10‐min walk, 800 m trot, 800 m relaxed canter), a conditioning canter at 16–22 s/200 m and a high‐intensity gallop at 13–15 s/200 m, followed by 30–40 min of cool‐down. To minimise differences in intensity, sessions were chosen to achieve similar target pace and distance within these ranges; during most sessions, commercial equine heart‐rate monitors were used to verify near‐maximal effort during the gallop. Flat race distances ranged from 1200 to 2600 m and were selected individually according to each horse's aptitude and training status.

Blood samples were obtained at rest (p0) and after a 30‐ to 40‐min recovery period (p1). For all stages, p0 was drawn in the morning after overnight stall rest and before any exercise on the sampling day. At T1 and T2, p0 preceded the scheduled high‐intensity gallop and was collected 5–10 days after the previous gallop session, within a programme in which gallop‐based high‐intensity work was typically scheduled every 5–10 days. In the race phase, p0 was collected on race day before any warm‐up, with race preparation planned so that the last high‐intensity training gallop occurred 5–10 days earlier. This schedule was intended to minimise carry‐over from the preceding strenuous session and to ensure that p0 represented a resting baseline for that day's effort. Previous equine studies have demonstrated robust mRNA changes within the first hour after exertion,^22^, ^23^, ^24^ with stress‐related genes such as HSPA6 peaking around 30 min^24^; accordingly, p1 was obtained 30–40 min after exercise to capture short‐lived gene‐expression responses that may decline at later time points.^22^, ^25^

The majority of horses participated in all three stages (T1, T2, R) (*n* = 26); however, a small number were excluded from one or more time points due to reasons such as early termination of training, sale of the animal or technical issues related to sample quality (e.g., RNA degradation or insufficient material for sequencing). In T1, a total of 65 samples were analysed, comprising 32 control samples collected pre‐exercise (p0) and 33 case samples obtained post‐exercise (p1). For T2, 75 samples were included, with 38 control samples collected before exercise and 37 case samples collected immediately after the session. From race, 64 samples were analysed, consisting of 32 controls (pre‐race) and 32 case samples (post‐race). The metadata of sample distribution are presented in Table S1. Venous blood was obtained aseptically from the jugular vein using sterile, single‐use needles. Samples were collected into EDTA tubes for haematological analysis and into plain tubes (Vacutainer, BD, USA) for serum biochemical evaluation. Additionally, excess blood was collected into PAXgene Blood RNA tubes (Qiagen, Germany) for transcriptomic analysis of gene expression.

### 2.3 RNA isolation and library preparation

Total RNA was isolated from whole‐blood specimens using the Mag‐Bind® PX Blood RNA Kit (Omega Bio‐tek) following the manufacturer's protocol. RNA integrity was evaluated on an Agilent 4200 TapeStation and concentrations quantified with a Qubit™ 4 Fluorometer. Libraries were prepared after rRNA depletion (Vazyme Ribo‐off™ rRNA Depletion Kit) using the VAHTS Universal V8 RNA‐seq Library Prep Kit (Vazyme) to generate strand‐specific libraries with ~300‐ to 400‐bp inserts. Libraries were normalised to equimolar concentration prior to sequencing.

### 2.4 Sequencing and primary QC

Sequencing was performed in paired‐end 150‐bp mode. Vendor metrics indicated a typical output of ~40 million reads per sample (≈20 million read pairs; ~6 Gb) with >90% bases ≥Q30. Adapter/quality trimming was applied by the vendor. Trimmed reads were aligned to the EquCab3.0 horse reference genome with HISAT2 v2.3.7 (splice‐aware defaults). The vendor provided primary gene‐level raw count matrices for downstream analyses (Table S2).

### 2.5 Data analysis and visualisation

All three contrasts—T1, T2 and R—were analysed with DESeq2 (R/Bioconductor). DESeq2's negative‐binomial framework with empirical dispersion estimation and Cook's distance outlier handling was used. Multiple testing was controlled with the Benjamini–Hochberg procedure and adjusted *p*‐values (padj) are reported. Repeated measurements were not modelled as dependent variables; each contrast was analysed as an independent two‐group comparison (CASE vs. CONTROL). Differentially expressed genes (DEGs) including baseMean, log2FoldChange, lfcSE (standard error of log2 fold change), stat (test statistic), *p*‐value and adjusted *p*‐value (padj) are presented in Tables [Link], [Link].

Counts were filtered to remove genes with zero counts across all samples, normalised by DESeq2 size factors and log‐transformed as log_2_(count/size factor + 1). For exploratory QC we computed sample–sample distance heatmaps (Euclidean distance on log_2_‐normalised counts) and principal component analysis (PCA). Group separation was visualised with 95% confidence ellipses. A priori outliers identified for QC views were screened by Mahalanobis distance in PC1–PC2 with a *χ* ^2^_2_ threshold of 9.21 (99%); flagged samples were excluded from analysis.

Genes meeting FDR < 0.05 and |log_2_FC| ≥ 1 were considered jointly significant for visualisation.

To summarise transcriptional programmes, we produced DEGs maps using the top 30 genes ranked by padj (preferring padj <0.05; if fewer than 30, filled by the next smallest padj). Values were row *z*‐scored to show relative up‐/down‐transcription per gene. Hierarchical clustering (average linkage, Euclidean distance) was applied to both genes and samples using SciPy and rendered in Matplotlib. MA plots: base mean (or average log_2_ expression) versus log_2_ fold change from DESeq2 results were drawn with Matplotlib. Volcano plots: log_2_FC versus −log_10_(FDR) from DESeq2 results. For readability with small FDRs, the *y*‐axis used adaptive caps (percentile‐based), with capped points indicated.

Detailed sample identifiers together with metadata and annotations that differentiate control and case groups for T1, T2 and the Rare presented in the Tables [Link], [Link].

Additionally, to verify breed‐ and sex‐dependent transcriptional differences within each condition, normalised count data were analysed using the limma–voom workflow in R.^26^ After voom transformation (log_2_‐counts per million with precision weights) we fitted linear models including breed and sex and provided Benjamini–Hochberg adjusted p‐values using empirical Bayes moderation. This dual‐step strategy leverages DESeq2 for broad discovery and limma‐voom for more precise subgroup modelling under potentially unbalanced sample designs (voom transformation + linear modelling + empirical Bayes moderation can handle smaller or imbalanced sample sizes more robustly). Log_2_ fold changes (log_2_FC) were derived for each comparison based on the top 30 DEGs previously identified by DESeq2. Limma‐voom was subsequently applied to these DEG‐defined gene sets to explicitly model repeated measurements from the same horse and to verify the consistency of effect directions and magnitudes under a linear modelling framework.

### 2.6 Protein–protein interaction network construction

Protein–protein interaction (PPI) networks were constructed using the STRING platform (version 12.0), with the minimum required interaction score set at 0.4 and visualised using Cytoscape (version 3.10.4).^27^ Only experimentally validated and database‐supported interactions were included in the final networks. To compare condition‐specific gene sets, we combined DEG‐derived STRING networks with Cytoscape's Merge tool. We used Intersection to obtain shared edges/nodes of all types of exertion (T1 ∩ T2 ∩ R) and Difference to obtain network‐specific elements (e.g., R − (T1 ∪ T2) for ‘Race‐only’).

For each primary network (T1, T2, Race) and each derived subnetwork (e.g., training‐only, race‐only), we ran Cytoscape Analyser as undirected graphs to compute global descriptors and degree‐based distributions.

### 2.7 Functional enrichment analysis of DEGs

To elucidate the biological relevance of the DEGs, we employed a dual enrichment approach using both g:Profiler and DAVID (Database for Annotation, Visualisation and Integrated Discovery) to improve annotation coverage and increase confidence in pathway‐level interpretations.

#### 2.7.1 g:Profiler analysis

Functional profiling was performed using g:Profiler (version e109_eg56_p17) [22], which enables gene set enrichment analysis across multiple ontology categories. DEGs were analysed separately for each exercise condition (T1, T2, R) against the stage‐specific universes defined as the genes that passed our QC filters and entered the DESeq2 models. The following annotation categories were assessed: Gene Ontology (GO) biological processes (BP), molecular functions (MF), cellular components (CC), Kyoto Encyclopedia of Genes and Genomes (KEGG) pathways and Human Phenotype Ontology (HP) terms.

Statistical significance was determined using a hypergeometric (Fisher's exact) test. To correct for multiple comparisons, the Benjamini–Hochberg false discovery rate (FDR) was applied, with terms considered significantly enriched at an adjusted *p*‐value (padj) < 0.05. This threshold was chosen based on standard practices in high‐throughput gene expression studies to balance sensitivity and control the FDR. Results were visualised using Manhattan plots directly generated by g:Profiler, where dot size indicates gene count and the vertical axis reflects significance (−log10 padj).

Functional enrichment analysis of PPI network's genes identified in Cytoscape was performed using the built‐in g:Profiler application to determine over‐represented BPs and pathways associated with each exercise condition. The enrichment was calculated using g:Profiler's hypergeometric test, applying the g_SCS multiple‐testing correction method to control for experiment‐wise FDR. Terms with an adjusted *p*‐value ≤0.05 were considered significant. Inferred electronic annotations were excluded to focus on experimentally supported gene–function associations.

Analyses included the following functional categories: GO BP, MF, HP as well as KEGG and Reactome pathway databases. To reduce redundancy among enriched terms, the Filter Enrichment Table tool in Cytoscape was applied with a redundancy cutoff of 0.5, merging terms with substantial overlap and retaining the most significant representative categories.

#### 2.7.2 DAVID analysis

To complement the g:Profiler results and verify consistency across platforms, functional annotation was also conducted in DAVID v6.8 [23] using the same DEG sets and condition‐specific backgrounds. Analysis categories included GO terms (BP, MF, CC), KEGG pathways, InterPro domains, UniProt keywords and SMART domains. Enrichment was assessed using modified Fisher's exact test (EASE score) and annotation clusters were automatically generated based on functional term co‐occurrence.

DAVID clusters with enrichment scores ≥1 were reported. Within clusters, only Benjamini‐significant terms were retained for display. Cluster bar charts (term gene counts with ES annotations) were produced in Python using Matplotlib/pandas.

## 3 RESULTS

### 3.1 Differentially expressed genes response to training session 1

**FIGURE 1:**
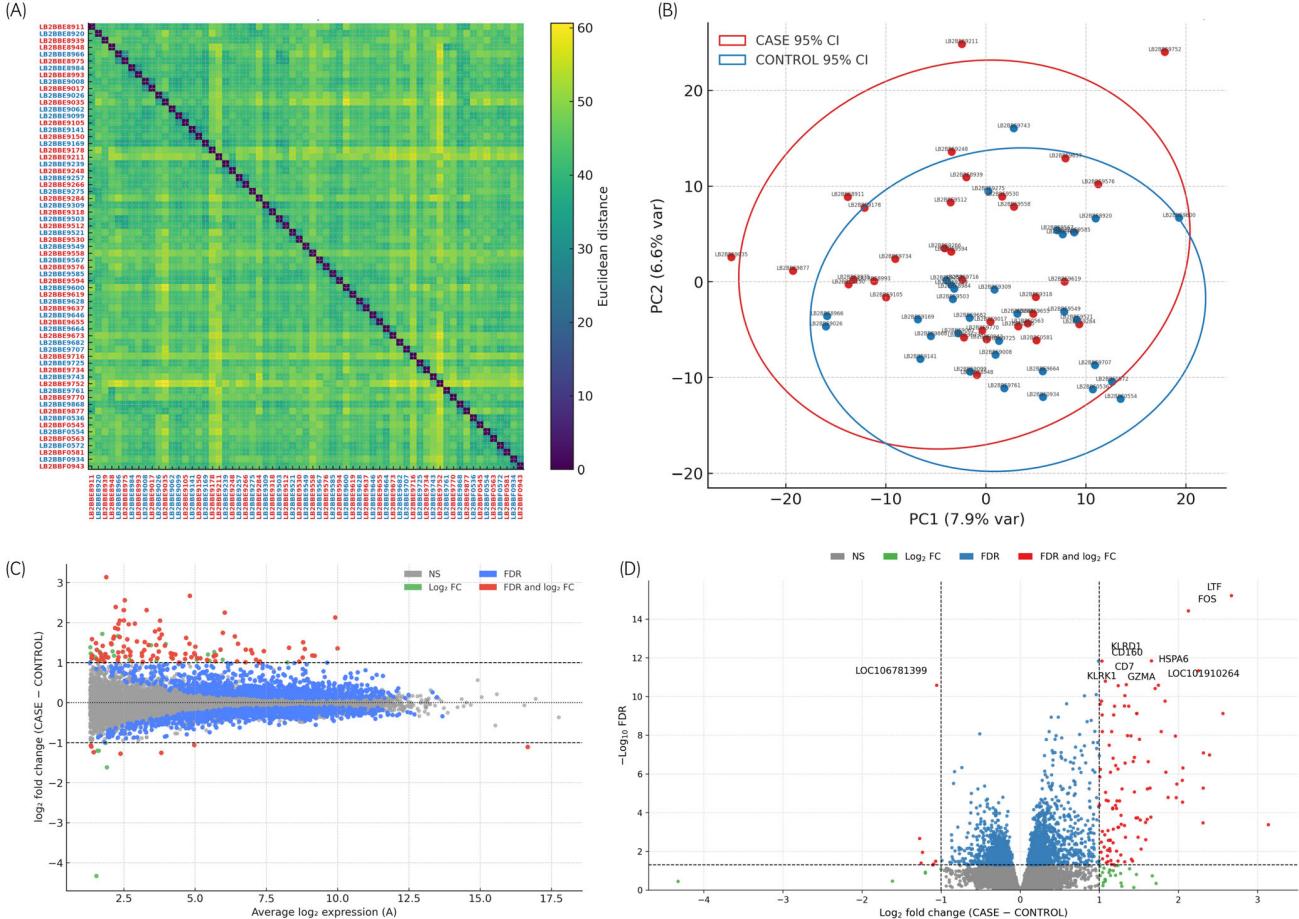
Global transcriptomic overview of horses at first training session (T1). (A) Sample‐to‐sample Euclidean distance heatmap based on the top 1000 highly variable genes (HVGs). Red labels: post‐exercise (case); blue labels: pre‐exercise (control). (B) Principal component analysis (PCA) of gene expression showing partial separation of case and control groups. Ellipses indicate 95% confidence intervals. (C) MA plot of differential expression results. Red: genes significant by both log2FC and FDR; blue: FDR only; green: log2FC only; grey: not significant. (D) Volcano plot highlighting significantly up‐ and down‐regulated genes (Benjamini–Hochberg adjusted). Selected immune‐related genes are labelled.

In T1 comparison, global structure was consistent with a phenotype effect overlaid on inter‐individual variability. The sample–sample heat‐map of Euclidean distances (top‐1000 HVGs; log_2_‐normalised and row‐*z*‐scored) showed tight control cohesion and a modest but reproducible increase in between‐group distances (mean ≈ 43.65) relative to within distances (post‐exercise ≈ 44.39, pre‐exercise ≈ 41.30; Δ_between–within ≈ +0.76) and PCA of the same matrix resolved PC1 = 7.9% and PC2 = 6.6% of the variance with partially separated 95% CIs for post‐exercise (CASE) and pre‐exercise (CONTROL) (Figure 1A,B). DESeq2 identified 1983/21,250 transcripts at FDR < 0.05, of which 115 also met |log_2_FC| ≥ 1 (108 up, 7 down).

The MA and volcano plots (Figure 1C,D) highlighted a compact set of strongly induced genes. The leading signals by adjusted p‐value included *LTF* (log_2_FC = 2.67), *HSPA6* (2.25), *FOS* (2.13), *KLRD1* (1.66), *KLRK1* (1.34), *CD7* (1.08) and *CD160* (1.03), alongside a notable down‐regulated locus *LOC106781399* (−1.06).

**FIGURE 2:**
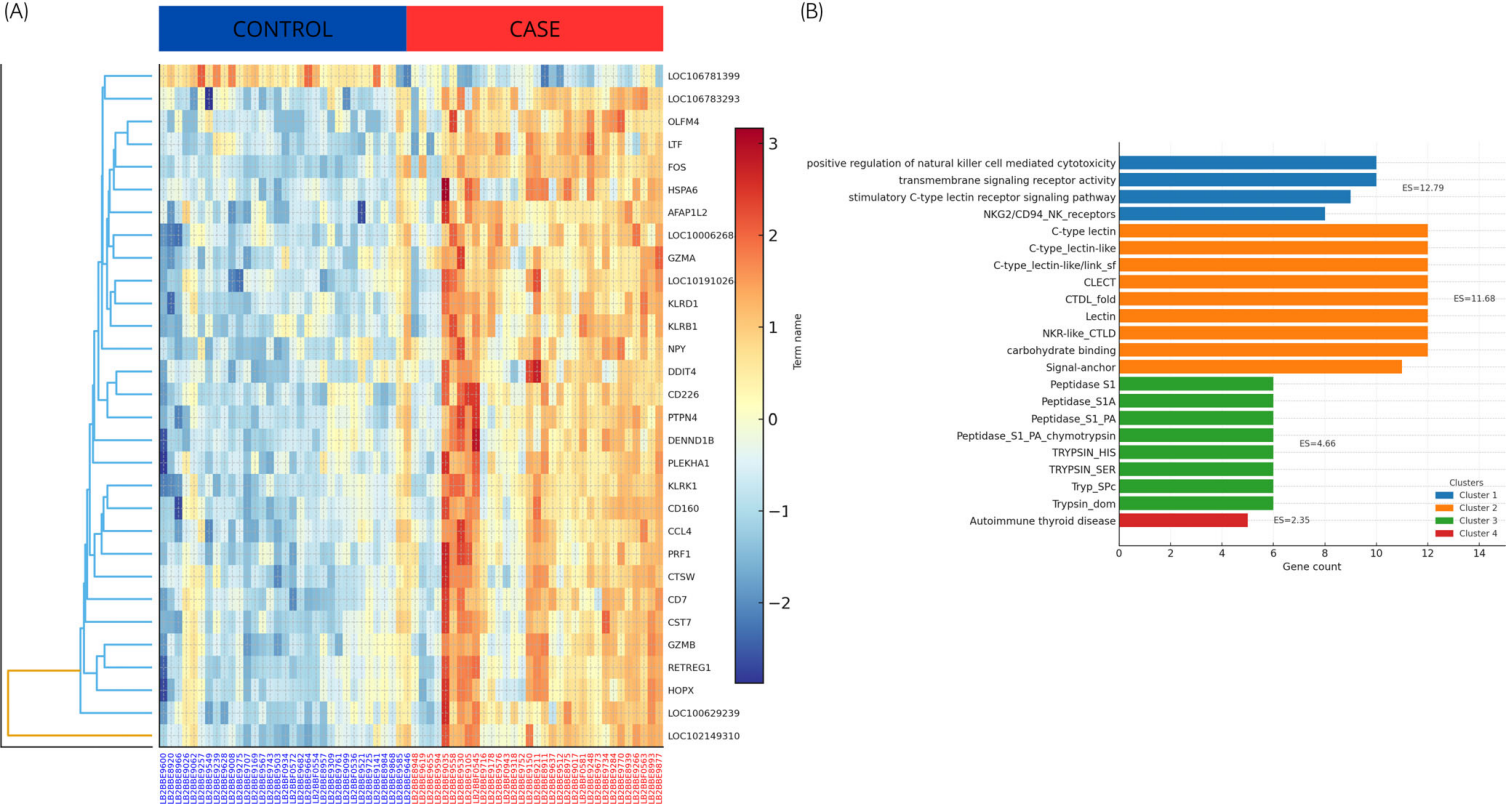
Transcriptional responses following the first training session (T1). (A) Heatmap of the top 30 DEGs identified at T1. Gene expression values (row‐wise *z*‐score of log_2_‐normalised counts) are colour‐scaled (blue = low expression; red = high expression). Columns represent individual horse samples, grouped by condition (blue = control; red = case) and hierarchically clustered by genes expression. (B) Functional enrichment of over‐expressed genes based on DAVID annotation clustering. Bars represent gene counts per cluster, coloured by annotation group, with enrichment scores labelled. DEGs, differentially expressed genes.

A heat‐map of the top 30 DEGs revealed a single dominant module that cleanly follows the phenotype split, composed of natural killer (NK)/cytotoxic and activation signatures—*KLRK1/KLRD1*, *PRF1*, *GZMA/GZMB*, *CTSW*, *CST7*, *CCL4*, *CD226*, *CD7*, *CD160* and immediate‐early/stress genes (*FOS*, *DDIT4*) with uniformly higher relative transcription in post‐exercise samples (Figure 2A).

Functional profiling agreed in terms of over‐expressed genes: g:Profiler returned significant GO/KEGG terms for NK‐cell receptor signalling, C‐type lectin receptor pathways and immune activation, while DAVID produced high‐scoring annotation clusters (e.g., ES ≈ 12.8 for NK/C‐type lectin receptor terms; ES ≈ 11.7 for carbohydrate‐binding/lectin families; and a serine‐protease/trypsin domain cluster, ES ≈ 4.7) (Figure 2B). In addition, HP terms related to erythrocyte morphology/fragility appeared in g:Profiler (Table 1), largely driven by the red‐cell genes *SLC4A*, *RHAG*, *SPTA1* and *EPB42* captured in the DEG lists.

**TABLE 1:** Terms identified in the functional enrichment analysis of over‐expressed genes at T1. Padj values shown are g SCS‐adjusted hypergeometric p‐values.

Term ID	Term name	Padj	Intersecting genes
GO:0170014	Ankyrin‐1 complex	7.02E‐08	*SLC4A1*, *AQP1*, *KLRF1*, *IFITM10*
HP:0004444	Spherocytosis	1.72E‐06	*SLC4A1*, *KLRF1*, *HEMGN*, *IFITM10*
GO:0032394	MHC class Ib receptor activity	1.57E‐05	*GIPR*, *CCL4*, *AFAP1L2*
HP:0005502	Increased red cell osmotic fragility	2.41E‐05	*SLC4A1*, *KLRF1*, *HEMGN*, *IFITM10*
HP:0020054	Abnormal erythrocyte physiology	3.47E‐05	*SLC4A1*, *KLRF1*, *HEMGN*, *IFITM10*
KEGG:05332	Graft‐versus‐host disease	2.65E‐04	*LOC100050711*, *LTF*, *NPY*, *AFAP1L2*
HP:0025546	Abnormal mean corpuscular haemoglobin concentration	2.87E‐04	*SLC4A1*, *KLRF1*, *HEMGN*, *IFITM10*
HP:0005525	Spontaneous haemolytic crises	7.69E‐04	*SLC4A1*, *KLRF1*, *HEMGN*
KEGG:04650	Natural killer cell‐mediated cytotoxicity	7.73E‐04	*LOC100050711*, *LTF*, *GIPR*, *NPY*, *AFAP1L2*
HP:0001923	Reticulocytosis	5.19E‐03	*SLC4A1*, *KLRF1*, *HEMGN*, *IFITM10*
GO:0042269	Regulation of natural killer cell‐mediated cytotoxicity	5.53E‐03	*CDC42EP2*, *GIPR*, *CCL4*, *AFAP1L2*
HP:0004446	Stomatocytosis	7.90E‐03	*SLC4A1*, *HEMGN*, *IFITM10*
GO:0002376	Immune system process	7.91E‐03	*CXCL8*, *SLC4A1*, *LOC100060505*, *LOC111772091*, *GABARAPL1*, *LOC100062688*, *FOS*, *OLFM4*, *LOC100069985*, *CDC42EP2*, *KLRF1*, *HEMGN*, *GIPR*, *ADGRG1*, *KLRK1*, *NPY*, *CCL4*, *ECATH‐2*, *AFAP1L2*
HP:0100724	Hypercoagulability	9.86E‐03	*SLC4A1*, *KLRF1*, *HEMGN*
HP:0025548	Increased mean corpuscular haemoglobin concentration	1.47E‐02	*SLC4A1*, *KLRF1*, *HEMGN*
GO:0042267	Natural killer cell‐mediated cytotoxicity	2.54E‐02	*CDC42EP2*, *GIPR*, *CCL4*, *AFAP1L2*
GO:0035379	Carbon dioxide transmembrane transporter activity	2.98E‐02	*AQP1*, *IFITM10*
KEGG:04612	Antigen processing and presentation	3.10E‐02	*LTF*, *NPY*, *AFAP1L2*
HP:0004312	Abnormal reticulocyte morphology	3.41E‐02	*SLC4A1*, *KLRF1*, *HEMGN*, *IFITM10*

Following the T1, several genes were identified significantly down‐regulated (*n* = 7). Three of them were identified as known locus: 28S ribosomal RNA (*RN28S*), *CCL8* (immune signalling) and protein phosphatase 2 regulatory subunit Bgamma (*PPP2R2C*) (Table 2). For complete DAVID‐based annotation for significantly over‐ and under‐expressed genes at T1, see Table S9.

**TABLE 2:** Functional annotation summary of significantly under‐expressed genes after T1 identified in DAVID analysis.

Gene name	GO biological process	GO molecular function	KEGG pathways
28S ribosomal RNA(RN28S)	—	Structural constituent of ribosome	Ribosome biogenesis in eukaryotes
C—C motif chemokine ligand 8 (CCL8)	Inflammatory response; immune response; chemokine‐mediated signalling pathway; positive regulation of cell migration	Chemokine activity; CCR chemokine receptor binding	Cytokine–cytokine receptor interaction; chemokine signalling pathway
Protein phosphatase 2 regulatory subunit Bgamma (PPP2R2C)	—	Protein phosphatase regulator activity	mRNA surveillance pathway, sphingolipid signalling pathway, PI3K‐Akt signalling pathway, AMPK signalling pathway, adrenergic signalling in cardiomyocytes, Hippo signalling pathway, Tight junction, T‐cell receptor signalling pathway, dopaminergic synapse, Chagas disease, hepatitis C, human papillomavirus infection

### 3.2 Differentially expressed genes response to training session 2

**FIGURE 3:**
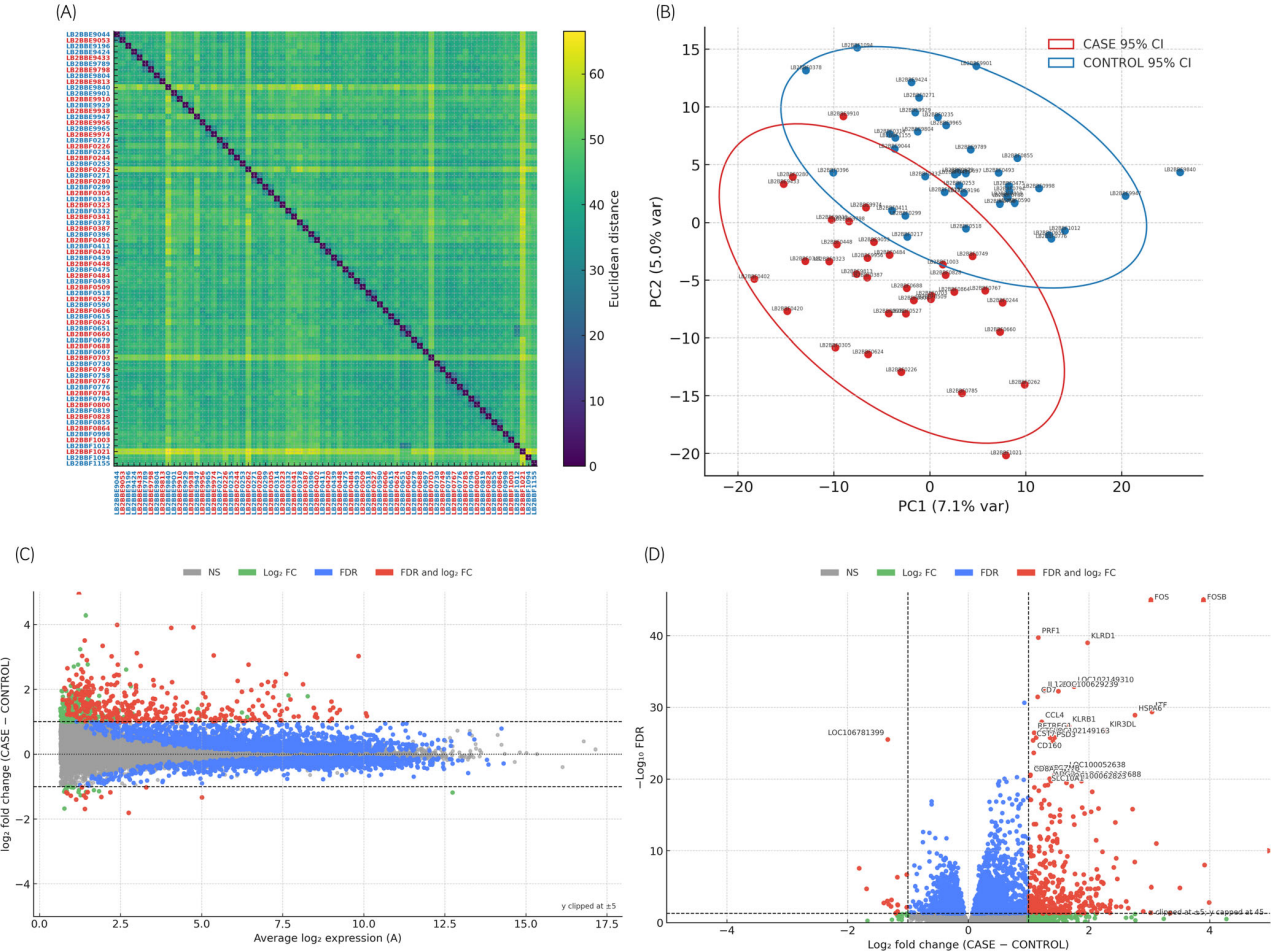
Global transcriptomic overview of horses at second training session (T2). (A) Sample‐to‐sample Euclidean distance heatmap based on the top 1000 highly variable genes (HVGs). Red labels: post‐exercise (case); blue labels: pre‐exercise (control). (B) Principal component analysis (PCA) of gene expression showing partial separation of case and control groups. Ellipses indicate 95% confidence intervals. (C) MA plot of differential expression results. Red: genes significant by both log2FC and FDR; blue: FDR only; green: log2FC only; grey: not significant. (D) Volcano plot highlighting significantly up‐ and down‐regulated genes (Benjamini–Hochberg adjusted). Selected immune‐related genes are labelled.

To assess the effects of repeated exercise, transcriptomic changes were analysed after the T2, aiming to capture adaptive molecular responses. Global QC indicated a reproducible case–control effect layered on inter‐individual variability. The sample–sample Euclidean‐distance heat map showed a between‐group mean of 40.43 (±5.30) versus within‐group means of 40.54 (±6.07, post‐exercise) and 38.26 (±3.75, pre‐exercise), that is, a modest excess of between‐group distance (Δ ≈ +1.16) with tighter cohesion among pre‐exercise samples (Figure 3A). PCA of the same matrix resolved PC1 = 7.1% and PC2 = 5.0% of variance with partially separated 95% ellipses (Figure 3B), reflecting a phenotype shift accompanied by higher dispersion within post‐exercise samples. DESeq2 identified 3700/24,612 transcripts at FDR <0.05, of which 375 also met |log_2_FC| ≥ 1 (360 up, 15 down).

**FIGURE 4:**
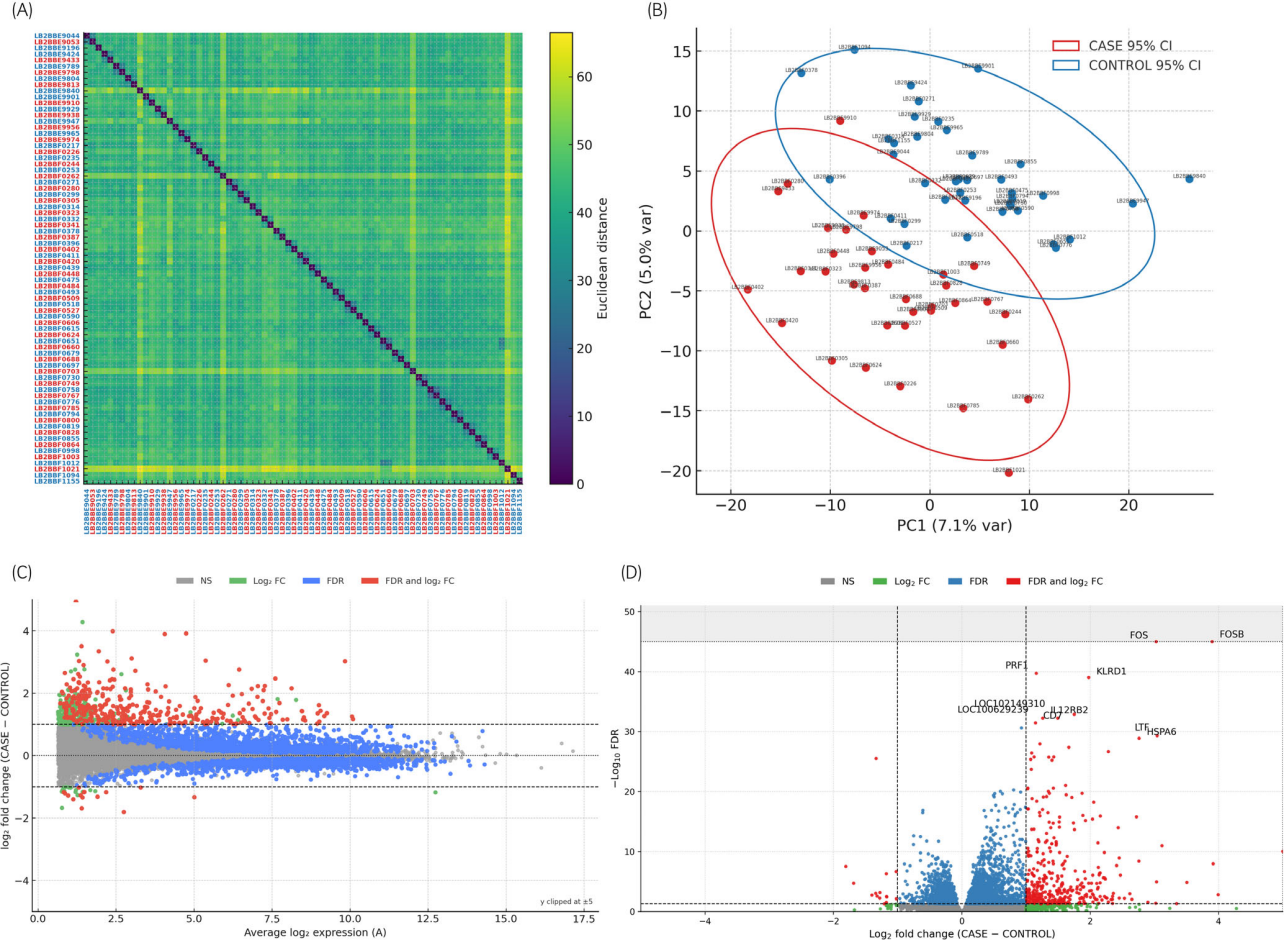
Transcriptional responses following the second training session (T2). (A) Heatmap of the top 30 DEGs identified at T2. Gene expression values (row‐wise *z*‐score of log_2_‐normalised counts) are colour‐scaled (blue = low expression; red = high expression). Columns represent individual horse samples, grouped by condition (blue = control; red = case) and hierarchically clustered by genes expression. (B) DAVID annotation clusters of over‐expressed genes at T2. Bar plots by gene count, coloured by cluster, enrichment score labelled. (C) T2‐specific subnetwork identified by Cytoscape Merge tool. The portion of the PPI network present exclusively in the T2 gene set and absent from the T1 and Race. Nodes represent differentially expressed genes; node size reflects degree (number of interactions) and node colour indicates direction and magnitude of expression change (red: over‐expressed, blue: under‐expressed). DEGs, differentially expressed genes; PPI, protein–protein interaction.

The MA and volcano plots (Figure 3C,D) highlighted immediate‐early and cytotoxic/NK programmes with large effect sizes, including *FOS* (log_2_FC = 3.03), *FOSB* (3.90), *HSPA6* (2.76), *LTF* (3.04), *KLRD1/CD94* (1.98), *KLRK1/NKG2D* (1.43), *KLRB1* (1.67), *PRF1* (1.16), *GZMB* (1.43), *GZMA* (1.35), *CCL4* (1.22), *CD7* (1.15) and *CD160* (1.08), alongside with a recurrent down‐regulated, uncharacterised locus LOC106781399. A heat map of the top 30 DEGs revealed a dominant co‐regulated module with higher relative transcription in post‐exercise samples that tracks the column grouping (CONTROL left, CASE right) and is enriched for NK/cytotoxic and activation genes (e.g., *KLRK1/KLRD1*, *PRF1*, *GZMB/GZMA*, *CCL4*, *CD7*) (Figure 4A).

At T2, functional enrichment analysis revealed overrepresentation of immune, erythroid and developmental pathways. The most significant terms included ‘cell killing’ (GO:0001906, *p* = 2.17 × 10^−5^), ‘NK cell‐mediated cytotoxicity’ (KEGG:04650, *p* = 7.21 × 10^−5^) and ‘immune system process’ (GO:0002376, *p* = 1.72 × 10^−4^). Erythroid‐related categories such as ‘Spherocytosis’ (HP:0004444, *p* = 4.31 × 10^−4^) and ‘Ankyrin‐1 complex’ (GO:0170014, *p* = 1.28 × 10^−3^) were also enriched, along with developmental terms including ‘developmental process’ (GO:0032502, *p* = 1.08 × 10^−4^) and ‘anatomical structure development’ (GO:0048856, *p* = 5.83 × 10^−4^) (Table 3).

**TABLE 3:** Terms identified in the functional enrichment analysis of over‐expressed genes at T2.

Term ID	Term name	Padj	Intersecting genes
GO:0001906	Cell killing	2.17E‐05	*LOC100630862*, *RBP1*, *KLRB1*, *ZNF704*, *AFAP1L2*, *HOPX*, *FBN1*, *MS4A3*, *SOBP*, *TIMP4*, *IL13*
KEGG:05332	Graft‐versus‐host disease	2.25E‐05	*LOC100630862*, *PREX2*, *HOPX*, *LCN2*, *TIMP4*, *CST7*
GO:0005615	Extracellular space	3.23E‐05	*RSPH6A*, *9796.ENSECAP00000000043*, *OLFM4*, *BBOF1*, *SPTA1*, *AFAP1L2*, *LAYN*, *PTK6*, *AZU1*, *FHDC1*, *IL22RA1*, *LOC102149205*, *ID1*, *SNAI1*, *LCN2*, *SOBP*, *GNLY*, *MYO18B*, *NEFL*, *TIMP4*, *HAVCR2*, *IL13*, *GREM1*, *C5H1orf21*, *CDC42EP2*, *TLCD4*, *LOC100051420*, *C30H1orf115*, *NEURL1B*
GO:0005102	Signalling receptor binding	3.93E‐05	*CNTFR*, *LOC100054999*, *ZDHHC1*, *GABARAPL1*, *BBOF1*, *KLRB1*, *SPTA1*, *ZNF704*, *ANKRD34A*, *IGF2*, *PTK6*, *FHDC1*, *HOPX*, *IL22RA1*, *VIL1*, *ID1*, *LCN2*, *CRISP2*, *CTSW*, *SOBP*, *GNLY*, *NEFL*, *TIMP4*, *EGFL7*, *GREM1*, *CDC42EP2*, *NEURL1B*, *NPY*, *CCL5*
KEGG:04650	Natural killer cell‐mediated cytotoxicity	7.21E‐05	*LOC100630862*, *PREX2*, *KLRB1*, *HOPX*, *LCN2*, *LOC100068447*, *TIMP4*, *CST7*
GO:0032502	Developmental process	1.08E‐04	*CNTFR*, *LOC100054358*, *ZDHHC1*, *FGF5*, *MYOZ3*, *LOC100068926*, *LOC106781485*, *FRMD4A*, *SLC9B1*, *FNDC1*, *CD8A*, *LOC100069985*, *EEF1A2*, *KLRF1*, *BBOF1*, *TRIM10*, *GYPA*, *KLRB1*, *AANAT*, *ZNF704*, *ANKRD34A*, *AFAP1L2*, *IFITM10*, *HSPA6*, *ARHGEF38*, *DDIT4L*, *PTK6*, *LOC100061252*, *FHDC1*, *BSPRY*, *LOC111772192*, *IL22RA1*, *ARHGAP33*, *TCN1*, *ID1*, *LCN2*, *GNRH1*, *SCN2A*, *CRYBG2*, *ALAS2*, *MS4A3*, *CXCL1*, *LOC111767409*, *PHEX*, *CA1*, *GNLY*, *MYO18B*, *TMEM108*, *NEFL*, *TIMP4*, *HAVCR2*, *EGFL7*, *IL13*, *ELOVL6*, *LOC106781721*, *GREM1*, *C5H1orf21*, *COL2A1*, *IFNG*, *DEPDC4*, *DDC*, *LOC100051420*, *SEMA3E*, *NKD1*, *ADD2*, *NEURL1B*, *SV2B*
GO:0009605	Response to external stimulus	1.30E‐04	*LOC100630862*, *ARR3*, *LOC100050711*, *LOC100068926*, *RSPH6A*, *LOC100064703*, *EDAR*, *LOC100069985*, *RBP1*, *BBOF1*, *KLRB1*, *ZNF704*, *ANKRD34A*, *AFAP1L2*, *IFITM10*, *CD160*, *HSPA6*, *PTK6*, *HOPX*, *VIL1*, *ID1*, *FBN1*, *MS4A3*, *SOBP*, *PHEX*, *TIMP4*, *EGFL7*, *IL13*, *GREM1*, *C5H1orf21*, *PLEKHH2*, *LOC100051420*, *C30H1orf115*, *NKD1*, *NEURL1B*
GO:0002376	Immune system process	1.72E‐04	*LOC100630862*, *LOC100054358*, *ARR3*, *LOC100064703*, *GABARAPL1*, *SLC9B1*, *OBSCN*, *LOC100069985*, *EEF1A2*, *RBP1*, *BBOF1*, *TRIM10*, *KLRB1*, *ZNF704*, *ANKRD34A*, *AFAP1L2*, *IGF2*, *HOPX*, *IL22RA1*, *TCN1*, *ID1*, *LCN2*, *FBN1*, *CRISP2*, *MS4A3*, *FASLG*, *SOBP*, *PHEX*, *TIMP4*, *EGFL7*, *IL13*, *C5H1orf21*, *IFNG*, *DEPDC4*, *LOC100051420*, *CST7*, *NEURL1B*, *NPY*, *CCL5*
GO:0048018	Receptor ligand activity	1.92E‐04	*ZDHHC1*, *BBOF1*, *SPTA1*, *PTK6*, *FHDC1*, *VIL1*, *ID1*, *LCN2*, *GNLY*, *TIMP4*, *EGFL7*, *GREM1*, *CDC42EP2*, *NEURL1B*, *NPY*, *CCL5*
GO:0007154	Cell communication	3.93E‐04	*CNTFR*, *FAAH*, *LOC100630862*, *LOC100054999, ZDHHC1*, *FGF5*, *LOC100050711*, *RGS1*, *LOC100068926*, *LOC100064703*, *GABARAPL1*, *LOC106781485*, *SLC9B1*, *EDAR*, *KLRF1*, *BBOF1*, *GYPA*, *KLRB1*, *SPTA1*, *ADGRG1*, *ZNF704*, *AP1M2*, *ANKRD34A*, *AFAP1L2*, *CD160*, *HSPA6*, *ARHGEF38*, *IGF2*, *LOC111772352*, *PTK6*, *LOC100061252*, *FHDC1*, *HOPX*, *BSPRY*, *IL22RA1*, *VIL1*, *ARHGAP33*, *ID1*, *LCN2*, *UCN*, *CRISP2*, *CRYBG2*, *ALAS2*, *CXCL8*, *MS4A3*, *CXCL1*, *FASLG*, *LOC111767409*, *PHEX*, *GNLY*, *TMEM108*, *NEFL*, *TIMP4*, *EGFL7*, *INSRR*, *IL13*, *GREM1*, *COL2A1*, *LOC100065586*, *CDC42EP2*, *LTF*, *PLEKHH2*, *LOC100051420*, *CST7*, *C7H11orf42*, *TSPAN33*, *C30H1orf115*, *NKD1*, *NEURL1B*, *SV2B*, *NPY*, *CCL5*, *LOC111773919*
GO:0032394	MHC class Ib receptor activity	4.16E‐04	*KLRB1*, *ZNF704*, *HOPX*
HP:0004444	Spherocytosis	4.31E‐04	*LOC100054358*, *EEF1A2*, *TRIM10*, *CCL4*
GO:0048856	Anatomical structure development	5.83E‐04	*CNTFR*, *LOC100054358*, *ZDHHC1*, *FGF5*, *MYOZ3*, *LOC100068926*, *LOC106781485*, *FRMD4A*, *SLC9B1*, *FNDC1*, *CD8A*, *LOC100069985*, *EEF1A2*, *KLRF1*, *BBOF1*, *TRIM10*, *GYPA*, *AANAT*, *ZNF704*, *ANKRD34A*, *AFAP1L2*, *IFITM10*, *HSPA6*, *ARHGEF38*, *PTK6*, *LOC100061252*, *FHDC1*, *BSPRY*, *IL22RA1*, *ARHGAP33*, *TCN1*, *ID1*, *LCN2*, *GNRH1*, *SCN2A*, *CRYBG2*, *ALAS2*, *MS4A3*, *CXCL1*, *LOC111767409*, *PHEX*, *CA1*, *GNLY*, *TMEM108*, *NEFL*, *TIMP4*, *HAVCR2*, *EGFL7*, *IL13*, *LOC106781721*, *GREM1*, *C5H1orf21*, *COL2A1*, *IFNG*, *DEPDC4*, *DDC*, *LOC100051420*, *SEMA3E*, *NKD1*, *ADD2*, *NEURL1B*, *SV2B*
KEGG:05144	Malaria	1.21E‐03	*DDIT4*, *RBP1*, *KLRB1*, *ID1*, *TIMP4*
GO:0170014	Ankyrin‐1 complex	1.28E‐03	*LOC100054358*, *EEF1A2*, *CCL4*
GO:0023024	MHC class I protein complex binding	4.10E‐03	*SLC9B1*, *ZNF704*, *HOPX*
HP:0004312	Abnormal reticulocyte morphology	4.95E‐03	*LOC100630862*, *LOC100054358*, *EEF1A2*, *TRIM10*, *LCN2*, *TIMP4*, *CCL4*
HP:0005502	Increased red cell osmotic fragility	5.87E‐03	*LOC100054358*, *EEF1A2*, *TRIM10*, *CCL4*
HP:0020054	Abnormal erythrocyte physiology	8.42E‐03	*LOC100054358*, *EEF1A2*, *TRIM10*, *CCL4*
KEGG:05330	Allograft rejection	8.74E‐03	*LOC100630862*, *PREX2*, *LCN2*, *TIMP4*
GO:0141061	Disruption of cell in another organism	1.14E‐02	*LOC100630862*, *AFAP1L2*, *FBN1*, *TIMP4*, *IL13*
GO:0051240	Positive regulation of multicellular organismal process	1.44E‐02	LOC100068926, CD8A, TRIM10, KLRB1, ADGRG1, AANAT, ZNF704, AFAP1L2, HSPA6, PTK6, LOC100061252, VIL1, ID1, ALAS2, MS4A3, SOBP, RERG, PHEX, GNLY, TIMP4, C5H1orf21, PLEKHH2, NEURL1B, NPY
GO:0097059	CNTFR‐CLCF1 complex	1.67E‐02	*CNTFR*, *NPY*
KEGG:04940	Type I diabetes mellitus	1.77E‐02	*LOC100630862*, *PREX2*, *LCN2*, *TIMP4*
KEGG:04657	IL‐17 signalling pathway	1.78E‐02	*AZU1*, *ID1*, *SOBP*, *PHEX*, *TIMP4*
GO:0042116	Macrophage activation	2.14E‐02	*AFAP1L2*, *MS4A3*, *SOBP*, *TIMP4*, *C5H1orf21*, *LOC100051420*
GO:0001909	Leukocyte‐mediated cytotoxicity	3.94E‐02	*LOC100630862*, *RBP1*, *KLRB1*, *ZNF704*, *AFAP1L2*, *HOPX*, *MS4A3*
GO:0032536	Regulation of cell projection size	4.34E‐02	*FRMD4A*, *HSPA6*, *ALAS2*
HP:0005525	Spontaneous haemolytic crises	4.59E‐02	*LOC100054358*, *EEF1A2*, *TRIM10*
GO:0001774	Microglial cell activation	4.95E‐02	*AFAP1L2*, *TIMP4*, *C5H1orf21*, *LOC100051420*

*Note*: Padj values shown are g_SCS‐adjusted hypergeometric *p*‐values.

DAVID functional clustering (Figure 4B) identified groups associated with transmembrane receptor signalling and C‐type lectin receptor pathways (ES = 11.43), as well as clusters related to peptidase activity (ES = 3.51), chemokine signalling (ES = 2.87) and blood microparticle formation (ES = 1.08).

DAVID annotation also highlighted a small set of down‐regulated transcripts at T2. These include *CCL8*, a chemokine involved in inflammatory/immune responses and chemokine‐mediated cell migration, *ARHGAP44*, a Rho GTPase‐activating protein linked to actin‐cytoskeleton organisation, negative regulation of Rac signalling and modulation of synaptic receptor trafficking; *OR52B2*, an olfactory G protein‐coupled receptor signalling pathway GPCR) (mapped to GPCR signalling/olfactory transduction); *SALL3*, an RNA Pol II transcription factor (sequence‐specific DNA binding); and *UPK3B*, a urothelial membrane structural component (Table 4).

**TABLE 4:** Functional annotation of significantly under‐expressed genes at T2.

Gene name	GO biological process	GO molecular function	KEGG pathways
C—C motif chemokine ligand 8 (CCL8)	Inflammatory response, immune response, chemokine‐mediated signalling pathway, positive regulation of cell migration	Chemokine activity, CCR chemokine receptor binding	Cytokine–cytokine receptor interaction, chemokine signalling pathway
Rho GTPase activating protein 44 (ARHGAP44)	Signal transduction, regulation of actin cytoskeleton organisation, negative regulation of Rac protein signal transduction, positive regulation of GTPase activity, modulation of chemical synaptic transmission, regulation of dendritic spine morphogenesis, modification of dendritic spine, neurotransmitter receptor transport, endosome to postsynaptic membrane, regulation of neurotransmitter receptor transport, endosome to postsynaptic membrane	GTPase activator activity, protein binding, phospholipid binding, small GTPase binding	—
Olfactory receptor family 52 subfamily B member 2 (OR52B2)	G‐protein‐coupled receptor signalling pathway	G‐protein‐coupled receptor activity, olfactory receptor activity	Olfactory transduction
Spalt‐like transcription factor 3 (SALL3)	Regulation of transcription by RNA polymerase II	RNA polymerase II cis‐regulatory region sequence‐specific DNA binding, DNA‐binding transcription factor activity, RNA polymerase II specific	—
Uroplakin 3B(UPK3B)	—	—	—

Together, their reduced expression suggests a modest counter‐regulatory module, dampening chemokine‐driven recruitment (*CCL8*) and tuning cytoskeletal/GPCR/transcriptional programmes (*ARHGAP44*, *OR52B2*, *SALL3*, *UPK3B*)—that sits alongside, but is secondary to, the dominant NK/cytotoxic up‐regulation observed in T2. For complete DAVID‐based annotation for significantly over‐ and under‐expressed genes at T2, see Table S9.

#### 3.2.1 Functional landscape of the T2‐specific gene network

Figure 4C illustrates the gene interaction network of DEGs exclusively identified at T2. The T2‐exclusive gene set captured transcriptional signatures specific to chronic physiological adaptation. Functional enrichment revealed strong activation of growth factor receptor signalling, cytokine communication and neuroendocrine regulation (Table 5).

**TABLE 5:** Terms identified in the functional enrichment analysis of differentially expressed genes unique to T2 (mid‐season training).

Term id	Term name	Padj	Intersecting genes
GO:0048018	Receptor ligand activity	4.05E‐05	*UCN*, *PDGFB*, *VEGFC*, *FGF5*, *IFNG*, *FGF18*, *GNRH1*
KEGG:04630	JAK–STAT signalling pathway	8.79E‐04	*IL13*, *PDGFB*, *IL22RA1*, *IFNG*
GO:0070851	Growth factor receptor binding	1.80E‐03	*PDGFB*, *VEGFC*, *FGF5*, *FGF18*
GO:0008083	Growth factor activity	2.52E‐03	*PDGFB*, *VEGFC*, *FGF5*, *FGF18*
HP:0030338	Abnormal circulating gonadotropin concentration	2.83E‐03	*HFM1*, *PDGFB*, *GNRH1*, *MEIOB*, *ZFPM2*
GO:0140677	Molecular function activator activity	7.78E‐03	*UCN*, *PDGFB*, *VEGFC*, *FGF5*, *IFNG*, *FGF18*, *GNRH1*
KEGG:05200	Pathways in cancer	1.12E‐02	*IL13*, *PDGFB*, *VEGFC*, *FGF5*, *IFNG*
GO:0005515	Protein binding	1.43E‐02	*UCN*, *MPP2*, *SNAP25*, *IL13*, *INSRR*, *PDGFB*, *VEGFC*, *ADD2*, *IL22RA1*, *FGF5*, *IFNG*, *FGF18*, *LOC100068447*, *GNRH1*, *ZFPM2*
HP:0000141	Amenorrhoea	2.27E‐02	*HFM1*, *PDGFB*, *GNRH1*, *MEIOB*, *ZFPM2*
HP:0030346	Abnormal circulating follicle‐stimulating hormone concentration	2.46E‐02	*HFM1*, *PDGFB*, *MEIOB*, *ZFPM2*
GO:0038023	Signalling receptor activity	3.01E‐02	*UCN*, *INSRR*, *PDGFB*, *VEGFC*, *IL22RA1*, *FGF5*, *IFNG*, *FGF18*, *GNRH1*
GO:0060089	Molecular transducer activity	3.01E‐02	*UCN*, *INSRR*, *PDGFB*, *VEGFC*, *IL22RA1*, *FGF5*, *IFNG*, *FGF18*, *GNRH1*

*Note:* Padj values shown are g_SCS‐adjusted hypergeometric *p*‐values.

The most significant enrichment (*p* = 4.05 × 10^−5^) was observed for ‘receptor ligand activity’ (GO:0048018), driven by *PDGFB*, *VEGFC*, *FGF5*, *FGF18* and *IFNG*, forming a coordinated growth and cytokine signalling axis.

The ‘JAK–STAT signalling pathway’ (KEGG:04630; *IL13*, *PDGFB*, *IL22RA1*, *IFNG*; *p* = 8.79 × 10^−4^) emerged as a central regulatory axis within the T2‐exclusive network. Genes associated with cytokine, growth factor and interferon signalling were concurrently over‐expressed, including *IL13*, *IFNG* and *IL22RA1*.

Among genes identified only at T2 analysis also revealed hormonal and neuroendocrine regulation, with enriched terms such as ‘Abnormal circulating gonadotropin concentration’ (HP:0030338) and ‘Abnormal circulating FSH concentration’ (HP:0030346), driven by *GNRH1*, *ZFPM2*, *PDGFB* and *HFM1*. Additional expression of *UCN*, *ADD2*, *SNAP25* and *MPP2* was observed, along with *MPC1L*, *HFM1* and *MEIOB*.

### 3.3 Differentially expressed genes response to race

**FIGURE 5:**
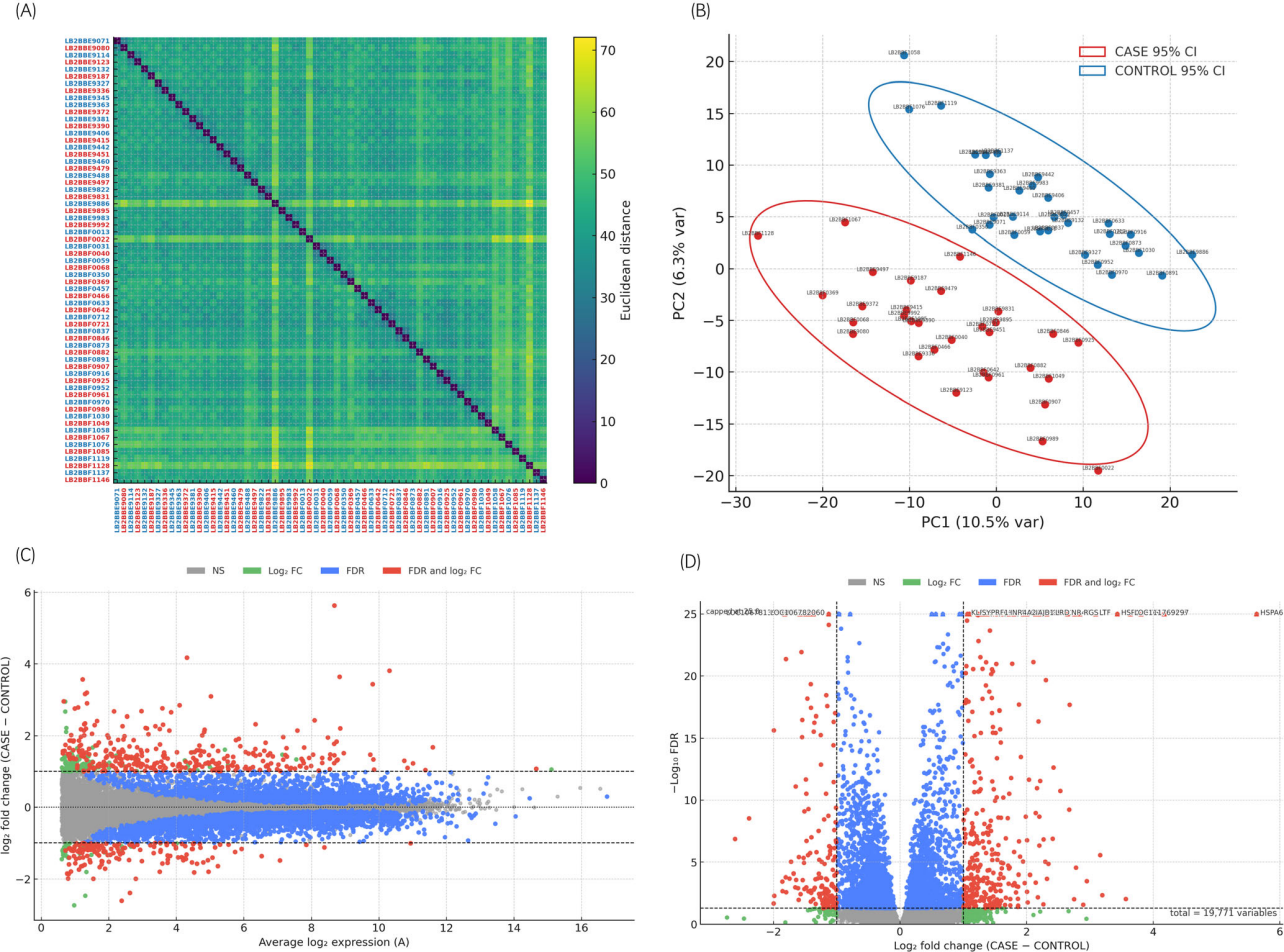
Global transcriptomic overview of horses at race (R). (A) Sample‐to‐sample Euclidean distance heatmap based on the top 1000 highly variable genes (HVGs). Red labels: post‐racr (case); blue labels: pre‐race (control). (B) Principal component analysis (PCA) of gene expression showing partial separation of case and control groups. Ellipses indicate 95% confidence intervals. (C) MA plot of differential expression results. Red: genes significant by both log2FC and FDR; blue: FDR only; green: log2FC only; grey: not significant. (D) Volcano plot highlighting significantly up‐ and down‐regulated genes (Benjamini–Hochberg adjusted). Selected immune‐related genes are labelled.

**FIGURE 6:**
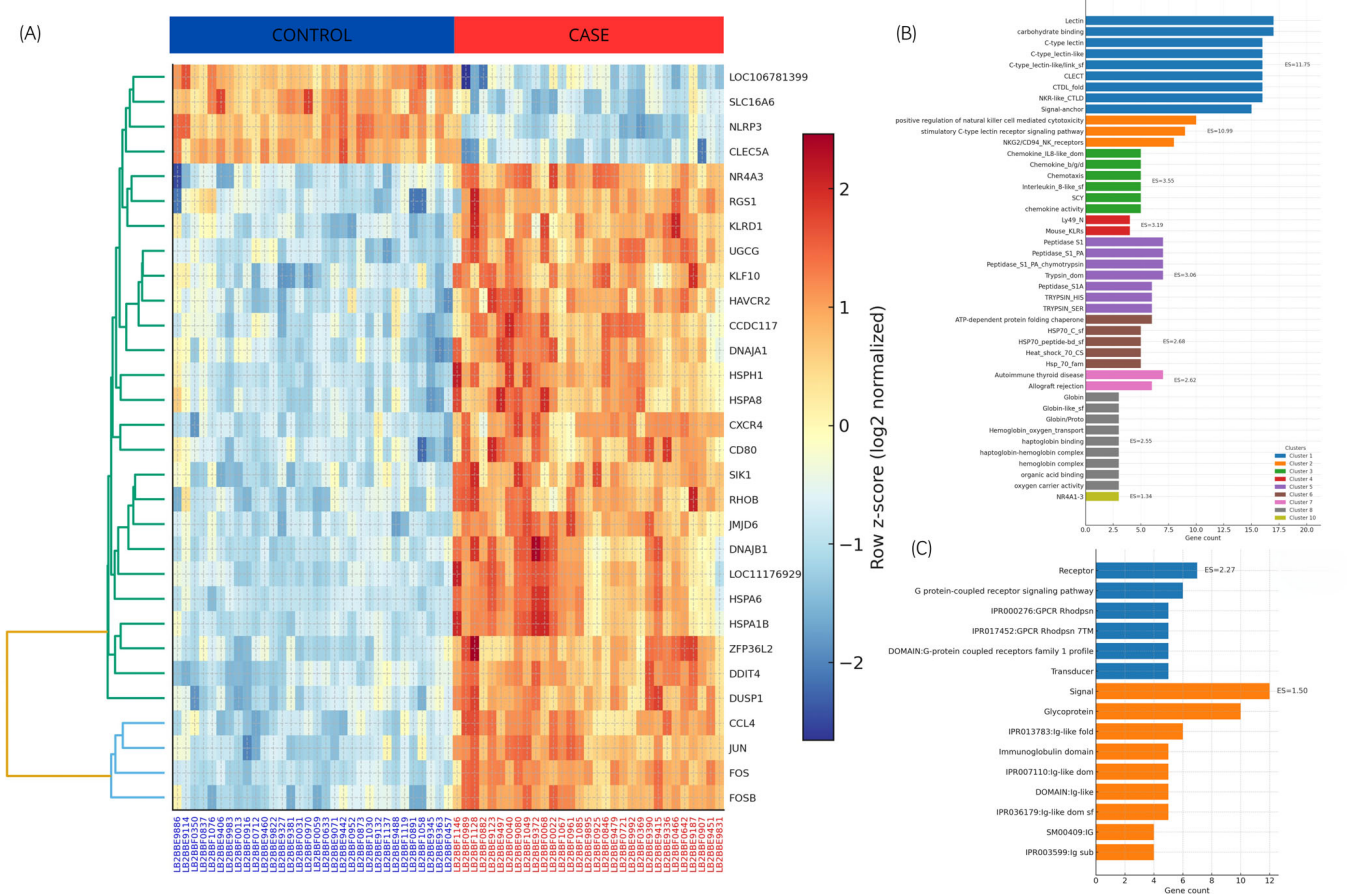
Transcriptional responses following race (R). (A) Heatmap of the top 30 DEGs identified at R. Gene expression values (row‐wise *z*‐score of log_2_‐normalised counts) are colour‐scaled (blue = low expression; red = high expression). Columns represent individual horse samples, grouped by condition (blue = control; red = case) and hierarchically clustered by genes expression. (B) DAVID annotation clusters of over‐expressed and (C) under‐expressed genes at R. Bar plots by gene count, coloured by cluster, enrichment score labelled. DEGs, differentially expressed genes.

In the Race response analysis, global QC indicated a clear phenotype signal on a stable cohort background: the sample–sample Euclidean map showed between‐group distances of 45.51 ± 5.84 versus within‐post‐race group: 43.73 ± 5.57 and within‐pre‐race: 42.54 ± 5.69 (Δ_between–within ≈ +2.40) and PCA resolved PC1 = 10.5% and PC2 = 6.3% with well‐separated 95% ellipses for post‐race (CASE) and pre‐race (CONTROL) sample (Figure 5A,B). DESeq2 identified 4358/19,771 genes at FDR < 0.05; 476 also satisfied |log_2_FC| ≥ 1 (325 up, 151 down). Leading signals combined immediate‐early and stress/chaperone responses with immune trafficking: *FOS* (log_2_FC = 3.81), *FOSB* (4.18), *JUN* (1.80), *DDIT4* (2.14), *HSPA6* (5.63), *HSPA1B* (3.43), *HSPH1* (2.20), *JMJD6* (1.38), *CCL4* (1.96) and *CXCR4* (1.30), while NLRP3 was down‐regulated (−1.49) (Figure 5C,D). The heat map of the top 30 DE genes cleanly followed the phenotype split and highlighted coordinated induction of HSP70 family chaperones (*HSPA6/HSPA1B/HSPA8/HSPH1/DNAJB1/DNAJA1*), immediate‐early transcription factors (*FOS/FOSB/JUN/NR4A3/KLF10*) and immune receptors/trafficking genes (*KLRD1*, *CLEC5A*, *HAVCR2*, *CXCR4*, *CCL4*), with *NLRP3* forming a counter‐trend of reduced expression (Figure 6A).

At Race, over‐expressed genes enrichment was dominated by stimulus/immune categories like ‘response to external stimulus’ (GO:0009605, *p* = 2.11E‐08), ‘immune system process’ (GO:0002376, *p* = 3.37E‐08) and ‘response to stress’ (GO:0006950, *p* = 4.18E‐05) with contributions from *PRF1*, *GNLY*, *CXCL8*, *TNFSF9*. Developmental terms were also prominent, including ‘circulatory system development’ (GO:0072359, *p* = 1.38E‐07), ‘developmental process’ (GO:0032502, *p* = 1.15E‐06), ‘cell differentiation’ (GO:0030154, p = 3.37E‐05), ‘vasculature development’ (GO:0001944, *p* = 2.39E‐04) and ‘blood vessel development/angiogenesis’ (GO:0001568, *p* = 5.80E‐04; GO:0001525, *p* = 1.33E‐03). Erythroid‐associated categories included ‘ankyrin‐1 complex’ (GO:0170014, *p* = 3.78E‐06), ‘Spherocytosis’ (HP:0004444, *p* = 3.34E‐04), ‘Increased red cell osmotic fragility’ (HP:0005502, *p* = 4.56E‐03) and ‘Abnormal reticulocyte morphology’ (HP:0004312, *p* = 4.66E‐02). Additional immune signalling terms were enriched, including ‘NK cell‐mediated cytotoxicity’ (KEGG:04650, *p* = 1.01E‐03) and ‘T‐cell activation’ (GO:0042110, *p* = 4.77E‐02) (Table 6).

**TABLE 6:** Terms identified in the functional enrichment analysis of over‐expressed genes at Race.

Term ID	Term name	Padj	Intersecting genes
GO:0009605	Response to external stimulus	2.11E‐08	*IL23R*, *GFI1*, *PRF1*, *AQP1*, *RGS1*, *GHSR*, *LOC100064703*, *DAPK2*, *EREG*, *LOC100069985*, *ANKRD42*, *KLRF1*, *TRIM10*, *ADAMTS14*, *CGA*, *AFAP1L2*, *EGR3*, *CD160*, *NEBL*, *IGF2*, *NKG7*, *PTK6*, *NFKBIA*, *TNFSF9*, *ALAS2*, *CXCL8*, *C23H9orf153*, *CA1*, *GNLY*, *PSD3*, *HSP90AA1*, *DUSP1*, *DUSP8*, *DCN*, *CKS2*, *LOC100051420*, *PARD6G*, *CXCR4*, *CST7*, *LMNA*
GO:0002376	Immune system process	3.37E‐08	*IL23R*, *GFI1*, *PRF1*, *SLC4A1*, *DAPK2*, *LOC100061672*, *EREG*, *JMJD6*, *CD8A*, *EEF1A2*, *KLRF1*, *TRIM10*, *GYPA*, *GIPR*, *ZFP36L2*, *ADGRG1*, *ADAMTS14*, *CGA*, *AFAP1L2*, *EGR3*, *NKG7*, *COL1A1*, *NFKBIA*, *NR4A3*, *SNAI1*, *TNFSF9*, *RETREG1*, *CXCL8*, *C23H9orf153*, *RERG*, *CA1*, *GNLY*, *PSD3*, *HSP90AA1*, *DUSP1*, *DUSP8*, *DCN*, *TUBB2A*, *SLCO4A1*, *DTHD1*, *SV2B*, *NPY*, *LMNA*, *CCL3*
GO:0072359	Circulatory system development	1.38E‐07	*AQP1*, *JMJD6*, *ANKRD42*, *ART4*, *CGA*, *AFAP1L2*, *JUN*, *NEBL*, *IGF2*, *PTK6*, *DCLK1*, *PNMA8C*, *NR4A2*, *NR4A3*, *TNFSF9*, *RETREG1*, *ALAS2*, *LOC111767409*, *MYO18B*, *TMEM108*, *ERMAP*, *DUSP8*, *ARL4A*, *LOC100051420*, *PARD6G*, *CST7*, *LMNA*
GO:0051240	Positive regulation of multicellular organismal process	1.06E‐06	*IL23R*, *AQP1*, *GHSR*, *EREG*, *DDIT4*, *LOC100056513*, *ZFP36L2*, *ADGRG1*, *ADAMTS14*, *HSPH1*, *CGA*, *AFAP1L2*, *IGF2*, *PNMA8C*, *COL1A1*, *TNFSF9*, *FASLG*, *C23H9orf153*, *RERG*, *CA1*, *PSD3*, *TMEM108*, *ERMAP*, *DUSP8*, *DCN*, *SLCO4A1*, *CKS2*, *LOC100051420*, *CST7*, *SV2B*
GO:0032502	Developmental process	1.15E‐06	*CNTFR*, *GFI1*, *DIXDC1*, *SLC4A1*, *AQP1*, *EFEMP1*, *PLS3*, *GHSR*, *EREG*, *JMJD6*, *KEL*, *CD8A*, *ANKRD42*, *KLRF1*, *GYPA*, *EPB42*, *LOC100056513*, *GIPR*, *ZFP36L2*, *ART4*, *ADGRG1*, *ADAMTS14*, *CGA*, *AFAP1L2*, *JUN*, *EGR3*, *CD160*, *NEBL*, *IGF2*, *LOC111772352*, *PTK6*, *DCLK1*, *PNMA8C*, *COL1A1*, *NR4A2*, *NR4A1*, *NR4A3*, *SNAI1*, *TNFSF9*, *RETREG1*, *ALAS2*, *CTSW*, *FASLG*, *LOC111767409*, *C23H9orf153*, *RERG*, *ADAMTS5*, *GNLY*, *PSD3*, *MYO18B*, *TMEM108*, *ERMAP*, *HAVCR2*, *KLF10*, *HSP90AA1*, *DUSP1*, *HBA2*, *DUSP8*, *DCN*, *ARL4A*, *TUBB2A*, *CDC42EP2*, *LTF*, *LOC100051420*, *PARD6G*, *CST7*, *LMNA*, *CCL3*
GO:0170014	Ankyrin‐1 complex	3.78E‐06	*SLC4A1*, *AQP1*, *GYPA*, *CCL4*
GO:0048856	Anatomical structure development	9.66E‐06	*CNTFR*, *GFI1*, *DIXDC1*, *SLC4A1*, *AQP1*, *EFEMP1*, *PLS3*, *GHSR*, *EREG*, *JMJD6*, *KEL*, *CD8A*, *ANKRD42*, *KLRF1*, *GYPA*, *EPB42*, *GIPR*, *ZFP36L2*, *ART4*, *ADGRG1*, *CGA*, *AFAP1L2*, *JUN*, *EGR3*, *CD160*, *NEBL*, *IGF2*, *LOC111772352*, *PTK6*, *DCLK1*, *PNMA8C*, *COL1A1*, *NR4A2*, *NR4A3*, *SNAI1*, *TNFSF9*, *RETREG1*, *ALAS2*, *CTSW*, *FASLG*, *LOC111767409*, *C23H9orf153*, *RERG*, *ADAMTS5*, *GNLY*, *PSD3*, *MYO18B*, *TMEM108*, *ERMAP*, *KLF10*, *HSP90AA1*, *DUSP1*, *DUSP8*, *DCN*, *ARL4A*, *TUBB2A*, *CDC42EP2*, *LTF*, *LOC100051420*, *PARD6G*, *CST7*, *LMNA*, *CCL3*
GO:0030154	Cell differentiation	3.37E‐05	*GFI1*, *DIXDC1*, *SLC4A1*, *EFEMP1*, *EREG*, *JMJD6*, *KEL*, *CD8A*, *ANKRD42*, *KLRF1*, *LOC100056513*, *GIPR*, *ZFP36L2*, *ART4*, *ADGRG1*, *ADAMTS14*, *CGA*, *JUN*, *EGR3*, *CD160*, *NEBL*, *IGF2*, *LOC111772352*, *PTK6*, *PNMA8C*, *COL1A1*, *NR4A2*, *NR4A1*, *NR4A3*, *SNAI1*, *RETREG1*, *ALAS2*, *CTSW*, *FASLG*, *LOC111767409*, *RERG*, *PSD3*, *ERMAP*, *HAVCR2*, *DUSP1*, *HBA2*, *DUSP8*, *ARL4A*, *TUBB2A*, *CDC42EP2*, *LTF*, *PARD6G*, *CST7*, *LMNA*, *CCL3*
GO:0006950	Response to stress	4.18E‐05	*IL23R*, *GFI1*, *PRF1*, *PLAT*, *SLC4A1*, *AQP1*, *GHSR*, *LOC100064703*, *EREG*, *LOC100069985*, *DDIT4*, *KLRF1*, *TRIM10*, *LOC100056513*, *GIPR*, *ADAMTS14*, *HSPH1*, *CD14*, *AFAP1L2*, *IFITM10*, *EGR3*, *NKG7*, *LOC111772352*, *PTK6*, *PNMA8C*, *NFKBIA*, *COL1A2*, *SNAI1*, *TNFSF9*, *ALAS2*, *CXCL8*, *FASLG*, *C23H9orf153*, *RERG*, *CA1*, *PSD3*, *TMEM108*, *HSP90AA1*, *DUSP1*, *DUSP8*, *DCN*, *C5H1orf21*, *ARL4A*, *LOC100051420*, *CXCR4*, *CST7*, *LMNA*
GO:0001944	Vasculature development	2.39E‐04	*AQP1*, *JMJD6*, *ART4*, *CGA*, *AFAP1L2*, *IGF2*, *PTK6*, *DCLK1*, *TNFSF9*, *RETREG1*, *LOC111767409*, *MYO18B*, *TMEM108*, *ERMAP*, *DUSP8*, *LOC100051420*, *CST7*, *LMNA*
HP:0004444	Spherocytosis	3.34E‐04	*SLC4A1*, *GYPA*, *ZFP36L2*, *CCL4*
GO:0032394	MHC class Ib receptor activity	3.95E‐04	*ADAMTS14*, *AFAP1L2*, *NFKBIA*
GO:0001568	Blood vessel development	5.80E‐04	*AQP1*, *JMJD6*, *ART4*, *CGA*, *AFAP1L2*, *IGF2*, *PTK6*, *DCLK1*, *TNFSF9*, *RETREG1*, *LOC111767409*, *MYO18B*, *TMEM108*, *ERMAP*, *LOC100051420*, *CST7*, *LMNA*
KEGG:05332	Graft‐versus‐host disease	6.70E‐04	*PRF1*, *LOC100050711*, *NFKBIA, RETREG1*, *DTHD1*
GO:0016477	Cell migration	7.10E‐04	*DIXDC1*, *PLAT*, *AQP1*, *DAPK2*, *ART4*, *ADAMTS14*, *CGA*, *EGR3*, *CD160*, *IGF2*, *LOC111772352*, *PTK6*, *MAFA*, *PNMA8C*, *TNFSF9*, *GNLY*, *MYO18B*, *TMEM108*, *ERMAP*, *DUSP8*, *ARL4A*, *LOC100051420*, *PARD6G*, *CST7*, *LMNA*, *CCL3*
GO:0009617	Response to bacterium	7.28E‐04	*IL23R*, *GFI1*, *AQP1*, *RGS1*, *LOC100064703*, *ADAMTS14*, *AFAP1L2*, *PTK6*, *TNFSF9*, *ALAS2*, *CXCL8*, *C23H9orf153*, *DUSP1*, *CKS2*, *LOC100051420*, *CXCR4*
KEGG:04650	Natural killer cell‐mediated cytotoxicity	1.01E‐03	*PRF1*, *LOC100050711*, *ADAMTS14*, *NFKBIA*, *RETREG1*, *TEAD2*, *DTHD1*
GO:0048646	Anatomical structure formation involved in morphogenesis	1.05E‐03	*AQP1*, *JMJD6*, *ART4*, *CGA*, *AFAP1L2*, *JUN*, *NEBL*, *IGF2*, *PTK6*, *PNMA8C*, *TNFSF9*, *RETREG1*, *ALAS2*, *LOC111767409*, *ADAMTS5*, *GNLY*, *MYO18B*, *TMEM108*, *ERMAP*, *LOC100051420*, *CST7*, *LMNA*
GO:0048870	Cell motility	1.23E‐03	*DIXDC1*, *PLAT*, *AQP1*, *DAPK2*, *ART4*, *ADAMTS14*, *CGA*, *EGR3*, *CD160*, *IGF2*, *LOC111772352*, *PTK6*, *MAFA*, *PNMA8C*, *NR4A2*, *TNFSF9*, *GNLY*, *MYO18B*, *TMEM108*, *ERMAP*, *DUSP8*, *ARL4A*, *HEMGN*, *LOC100051420*, *PARD6G*, *CST7*, *LMNA*, *CCL3*
GO:0001525	Angiogenesis	1.33E‐03	*AQP1*, *JMJD6*, *ART4*, *CGA*, *AFAP1L2*, *PTK6*, *TNFSF9*, *RETREG1*, *MYO18B*, *TMEM108*, *ERMAP*, *LOC100051420*, *CST7*, *LMNA*
GO:0040011	Locomotion	1.56E‐03	*AQP1*, *DAPK2*, *ART4*, *ADAMTS14*, *CGA*, *IGF2*, *PTK6*, *MAFA*, *PNMA8C*, *TNFSF9*, *FASLG*, *GNLY*, *MYO18B*, *TMEM108*, *DUSP8*, *ARL4A*, *HEMGN*, *LOC100051420*, *PARD6G*, *CST7*, *LMNA*, *CCL3*
GO:0048514	Blood vessel morphogenesis	2.33E‐03	*AQP1*, *JMJD6*, *ART4*, *CGA*, *AFAP1L2*, *PTK6*, *TNFSF9*, *RETREG1*, *LOC111767409*, *MYO18B*, *TMEM108*, *ERMAP*, *LOC100051420*, *CST7*, *LMNA*
GO:0043067	Regulation of programmed cell death	2.66E‐03	*CNTFR*, *PRF1*, *AQP1*, *DAPK2*, *SLFN14*, *ANKRD42*, *CD14*, *CGA*, *AFAP1L2*, *EGR3*, *LOC111772352*, *PTK6*, *PNMA8C*, *NR4A1*, *RETREG1*, *FASLG*, *CA1*, *TMEM108*, *ERMAP*, *ARL4A*, *CXCR4*, *CST7*, *LMNA*
HP:0200042	Skin ulcer	2.79E‐03	*GFI1*, *SLC4A1*, *GYPA*, *ZFP36L2*, *TGFB2*, *IGF2*, *NR4A1*, *C23H9orf153*, *ARL4A*
GO:0033993	Response to lipid	3.57E‐03	*IL23R*, *GFI1*, *AQP1*, *LOC100069985*, *GIPR*, *ADAMTS14*, *EGR3*, *PTK6*, *TNFSF9*, *LOC111767409*, *C23H9orf153*, *DUSP1*, *CKS2*, *LOC100051420*, *CST7*
GO:0023024	MHC class I protein complex binding	3.91E‐03	*CD8A*, *AFAP1L2*, *NFKBIA*
GO:1901700	Response to oxygen‐containing compound	3.95E‐03	*IL23R*, *GFI1*, *AQP1*, *GHSR*, *LOC100069985*, *ADGRG1*, *ADAMTS14*, *CD14*, *EGR3*, *SLC10A1*, *LOC111772352*, *PTK6*, *MAFA*, *TNFSF9*, *LOC111767409*, *C23H9orf153*, *PSD3*, *TMEM108*, *DUSP1*, *CKS2*, *LOC100051420*, *CST7*
GO:0009719	Response to endogenous stimulus	4.30E‐03	*AQP1*, *GHSR*, *LOC100069985*, *GIPR*, *ART4*, *ADGRG1*, *CD14*, *CGA*, *LOC111772352*, *PTK6*, *MAFA*, *DCLK1*, *NR4A3*, *TNFSF9*, *CRYBG2*, *CTSW*, *PSD3*, *MYO18B*, *ERMAP*, *LOC100051420*, *PARD6G*, *CST7*
HP:0005502	Increased red cell osmotic fragility	4.56E‐03	*SLC4A1*, *GYPA*, *ZFP36L2*, *CCL4*
KEGG:04210	Apoptosis	5.25E‐03	*PRF1*, *LOC100050711*, *ZFP36L2*, *CD14*, *RETREG1*, *PSD3*, *ARL4A*
GO:0043435	Response to corticotropin‐releasing hormone	5.92E‐03	*LOC111772352*, *PTK6*, *MAFA*
HP:0011407	Proportionate tall stature	6.53E‐03	*TGFB2*, *COL1A2*, *PSD3*, *HEMGN*
HP:0020054	Abnormal erythrocyte physiology	6.53E‐03	*SLC4A1*, *GYPA*, *ZFP36L2*, *CCL4*
GO:0048519	Negative regulation of biological process	1.09E‐02	*CNTFR*, *IL23R*, *GFI1*, *DIXDC1*, *PLAT*, *SLC4A1*, *AQP1*, *EFEMP1*, *RGS1*, *LOC100068926*, *GHSR*, *EREG*, *JMJD6*, *KEL*, *LOC100069985*, *ANKRD42*, *LOC100056513*, *GIPR*, *ART4*, *ADGRG1*, *ADAMTS14*, *CD14*, *CGA*, *AFAP1L2*, *EGR3*, *CD160*, *NEBL*, *IGF2*, *LOC111772352*, *PTK6*, *PNMA8C*, *NFKBIA*, *NR4A1*, *NR4A3*, *TNFSF9*, *RETREG1*, *CRYBG2*, *ALAS2*, *FASLG*, *C23H9orf153*, *RERG*, *ADAMTS5*, *GNLY*, *MYO18B*, *TMEM108*, *ERMAP*, *DUSP1*, *DCN*, *C5H1orf21*, *ARL4A*, *SLCO4A1*, *PARD6G*, *CST7*, *LMNA*, *CCL3*
GO:0005584	Collagen type I trimer	1.56E‐02	*IGF2*, *DCLK1*
GO:0097059	CNTFR‐CLCF1 complex	1.56E‐02	*CNTFR*, *SV2B*
GO:0032496	Response to lipopolysaccharide	1.91E‐02	*IL23R*, *GFI1*, *ADAMTS14*, *PTK6*, *TNFSF9*, *C23H9orf153*, *DUSP1*, *CKS2*, *LOC100051420*
KEGG:04657	IL‐17 signalling pathway	1.91E‐02	*CD14*, *BSPRY*, *TNFSF9*, *PSD3*, *CCL5*
GO:0030097	Haemopoiesis	1.99E‐02	*SLC4A1*, *JMJD6*, *CD8A*, *KLRF1*, *GIPR*, *ZFP36L2*, *ADGRG1*, *CGA*, *COL1A1*, *NR4A3*, *SNAI1*, *RERG*, *PSD3*, *TUBB2A*, *CDC42EP2*, *CST7*, *CCL3*
GO:0001816	Cytokine production	2.27E‐02	*IL23R*, *GHSR*, *LOC100061672*, *EREG*, *DDIT4*, *ADGRG1*, *ADAMTS14*, *HSPH1*, *AFAP1L2*, *C23H9orf153*, *CA1*, *DUSP1*, *SLCO4A1*, *CKS2*, *CST7*
KEGG:05144	Malaria	2.40E‐02	*SIK1*, *TRIM10*, *ADAMTS14*, *TNFSF9*
GO:0040012	Regulation of locomotion	2.43E‐02	*AQP1*, *DAPK2*, *ART4*, *ADAMTS14*, *IGF2*, *MAFA*, *PNMA8C*, *TNFSF9*, *GNLY*, *MYO18B*, *TMEM108*, *DUSP8*, *ARL4A*, *HEMGN*, *LOC100051420*, *PARD6G*, *CST7*, *CCL3*
KEGG:05418	Fluid shear stress and atherosclerosis	2.56E‐02	*PLAT*, *CD14*, *TGFB2*, *GNLY*, *PSD3*, *LOC100051420*
HP:0008887	Adipose tissue loss	2.56E‐02	*TGFB2*, *NR4A3*, *PSD3*, *ARL4A*, *HEMGN*
GO:0071363	Cellular response to growth factor stimulus	2.69E‐02	*GIPR*, *ART4*, *CD14*, *CGA*, *PTK6*, *DCLK1*, *NR4A3*, *TNFSF9*, *CTSW*, *PSD3*, *MYO18B*, *ERMAP*, *PARD6G*, *CST7*
GO:1903037	Regulation of leukocyte cell–cell adhesion	2.76E‐02	*ZFP36L2*, *ADGRG1*, *CGA*, *AFAP1L2*, *MAFA*, *COL1A1*, *C23H9orf153*, *SLCO4A1*, *CST7*, *CCL3*
HP:0040186	Maculopapular exanthema	3.40E‐02	*PRF1*, *SLC4A1*, *GYPA*, *ZFP36L2*
HP:0005525	Spontaneous haemolytic crises	3.72E‐02	*SLC4A1*, *GYPA*, *ZFP36L2*
HP:0004312	Abnormal reticulocyte morphology	4.66E‐02	*PRF1*, *SLC4A1*, *GYPA*, *ZFP36L2*, *RETREG1*, *CCL4*
GO:0042110	T‐cell activation	4.77E‐02	*JMJD6*, *CD8A*, *EEF1A2*, *GIPR*, *ZFP36L2*, *ADGRG1*, *CGA*, *AFAP1L2*, *COL1A1*, *C23H9orf153*, *SLCO4A1*, *CCL3*

*Note*: Padj values shown are g_SCS‐adjusted hypergeometric *p*‐values.

The DAVID functional cluster analysis supported these findings (Figure 6B), revealing the top clusters associated with lectin and carbohydrate‐binding pathways (ES = 11.75), positive regulation of NK cell‐mediated toxicity (ES = 10.99), chemokine and interleukin‐like domains (ES = 3.55) and peptidase and trypsin‐related families (ES ≈ 3.0). Additional clusters were linked to protein folding chaperones, globin and haemoglobin complexes and oxygen transport activity, indicating molecular signatures related to immune modulation, proteostasis and erythrocyte function at race. The down‐regulated gene set in the race condition formed two coherent annotation clusters (Figure 6C). Cluster 1 (ES = 2.27) comprises class‐A rhodopsin‐like GPCRs and related terms: G‐protein‐coupled receptor signalling pathway, InterPro GPCR rhodopsin 7TM domains, G‐protein‐coupled receptors family 1 profile and transducer, pointing to a concerted attenuation of receptor‐mediated environmental sensing/signalling. Cluster 2 (ES = 1.50) groups glycoprotein/Ig‐superfamily features: glycoprotein, Ig‐like fold/domain (InterPro/SMART) and related supersets suggesting a reduction in cell‐surface/secreted Ig‐like molecules and adhesion/communication modules. Consistently, g:Profiler recovered KEGG: Renin secretion as a significant term based on genes *SMIM5*, *LILRA* and *NLRP3* (padj = 2.3495 × 10^−2^), indicating broader dampening of select hormone/GPCR‐linked pathways. Together, these enrichments indicate that, alongside the robust induction of immediate‐early, chaperone and NK/trafficking programmes in Race, there is a concurrent suppression of GPCR‐driven signalling and Ig‐like/glycoprotein components. For complete DAVID‐based annotation for significantly over‐ and under‐expressed genes at R, see Table S9.

#### 3.3.1 Race‐specific transcriptional signatures reflect acute stress, immune activation and vascular Remodelling

**FIGURE 7:**
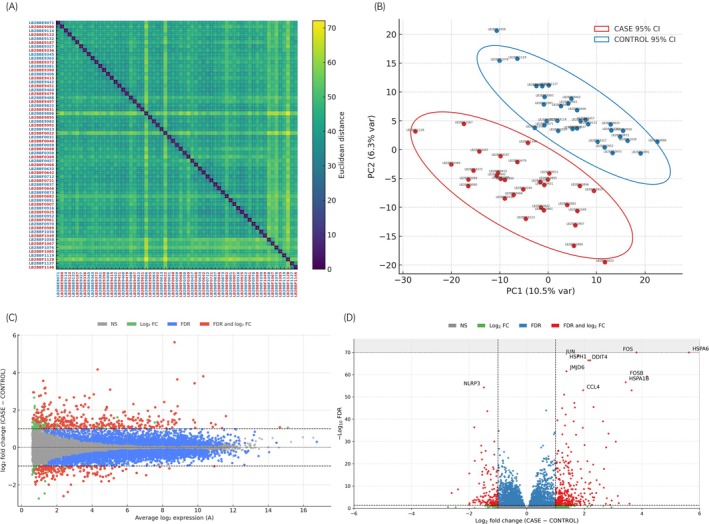
Race‐specific subnetwork identified by Cytoscape Merge tool. Portion of the largest network unique to the Race group and absent from the T1 and T2 networks (A). Isolated network of all down‐regulated genes within the Race‐specific module (B). Nodes represent differentially expressed genes; node size reflects degree (number of interactions) and node colour indicates relative expression (red: over‐expressed, blue: under‐expressed).

The R‐ exclusive gene network, specific to the race condition, represents the transcriptomic response to acute maximal exertion, integrating stress, immune, vascular and structural remodelling processes (Figure 7A). Unlike the training networks (T1, T2), the race response revealed broad activation of stress‐protective and repair pathways accompanied by selective suppression of inflammatory and structural genes. A full list of significantly enriched terms in the Race‐specific network is presented in Table 7.

**TABLE 7:** Terms identified from functional enrichment analysis of differentially expressed genes unique to Race.

Term ID	Term name	Padj	Intersecting genes
GO:0009605	Response to external stimulus	3.11E‐07	*GFI1*, *ADAMTS5*, *RGS1*, *HSP90AA1*, *DUSP1*, *DAPK2*, *EREG*, *TUBB2A*, *DDIT4*, *AMIGO2*, *PARD6G*, *HSPH1*, *TIGIT*, *EGR3*, *CAV1*, *HSPA6*, *NKG7*, *FMO1*, *KLF4*, *NFKBIA*
GO:0040011	Locomotion	1.77E‐06	*DUSP1*, *DAPK2*, *DCN*, *RHOB*, *AMIGO2*, *CXCR4*, *DNAJB1*, *TIGIT*, *JUN*, *EGR3*, *CAV1*, *FMO1*, *KLF4*, *NFKBIA*, *COL1A1*
GO:0072359	Circulatory system development	5.85E‐06	*ADAMTS5*, *DCN*, *RHOB*, *DDIT4*, *AMIGO2*, *CXCR4*, *DNAJB1*, *TIGIT*, *EGR3*, *CAV1*, *FMO1*, *KLF4*, *DCLK1*, *NFKBIA*
GO:0016477	Cell migration	6.27928E‐06	*DUSP1*, *DAPK2*, *DCN*, *RHOB*, *AMIGO2*, *CXCR4*, *DNAJB1*, *TIGIT*, *EGR3*, *CAV1*, *HSPA6*, *FMO1*, *MAFA*, *KLF4*, *NFKBIA*, *COL1A1*
GO:0048870	Cell motility	7.21589E‐06	*DUSP1*, *DAPK2*, *DCN*, *RHOB*, *AMIGO2*, *CXCR4*, *DNAJB1*, *TIGIT*, *JUN*, *EGR3*, *CAV1*, *HSPA6*, *FMO1*, *MAFA*, *KLF4*, *NFKBIA*, *COL1A1*
GO:0001944	Vasculature development	6.93E‐05	*DCN*, *RHOB*, *AMIGO2*, *CXCR4*, *TIGIT*, *EGR3*, *CAV1*, *FMO1*, *KLF4*, *DCLK1*, *NFKBIA*
GO:0035556	Intracellular signal transduction	8.56‐05	*CDKN1A*, *PREX2*, *DUSP2*, *DUSP1*, *DAPK2*, *DCN*, *RHOB*, *TUBB2A*, *DDIT4*, *AMIGO2*, *PARD6G*, *HSPH1*, *NLRP3*, *EGR3*, *CAV1*, *HSPA6*, *MAFA*, *KLF4*, *DCLK1*, *NFKBIA*
GO:0007154	Cell communication	1.34E‐04	*FAAH*, *CDKN1A*, *GFI1*, *PREX2*, *RGS1*, *DUSP2*, *DUSP1*, *DAPK2*, *DCN*, *EREG*, *RHOB*, *TUBB2A*, *DDIT4*, *AMIGO2*, *PARD6G*, *CXCR4*, *DNAJB1*, *HSPH1*, *NLRP3*, *TIGIT*, *EGR3*, *NOS3*, *CAV1*, *HSPA6*, *NKG7*, *FMO1*, *MAFA*, *KLF4*, *DCLK1*, *NFKBIA*
GO:0040012	Regulation of locomotion	2.10E‐04	*DUSP1*, *DAPK2*, *DCN*, *RHOB*, *AMIGO2*, *CXCR4*, *DNAJB1*, *JUN*, *EGR3*, *CAV1*, *FMO1*, *COL1A1*
GO:0043542	Endothelial cell migration	2.61E‐04	*DCN*, *RHOB*, *CXCR4*, *TIGIT*, *EGR3*, *CAV1*, *KLF4*
GO:0042981	Regulation of apoptotic process	3.70E‐04	*HSP90AA1*, *DAPK2*, *RHOB*, *DDIT4*, *PARD6G*, *CXCR4*, *DNAJB1*, *NLRP3*, *TIGIT*, *CAV1*, *MAFA*, *KLF4*, *NFKBIA*
GO:0051716	Cellular response to stimulus	4.14E‐04	*CDKN1A*, *GFI1*, *PREX2*, *RGS1*, *HSP90AA1*, *DUSP2*, *DUSP1*, *DAPK2*, *DCN*, *EREG*, *RHOB*, *TUBB2A*, *DDIT4*, *AMIGO2*, *PARD6G*, *CXCR4*, *PTGS2*, *DNAJB1*, *HSPH1*, *NLRP3*, *TIGIT*, *EGR3*, *HSPA1L*, *CAV1*, *HSPA6*, *NKG7*, *FMO1*, *MAFA*, *KLF4*, *DCLK1*, *NFKBIA*
GO:0006950	Response to stress	4.65E‐04	*CDKN1A*, *GFI1*, *ADAMTS5*, *HSP90AA1*, *EREG*, *RHOB*, *TUBB2A*, *AMIGO2*, *PARD6G*, *CXCR4*, *PTGS2*, *DNAJB1*, *HSPH1*, *NLRP3*, *EGR3*, *HSPA1L*, *CAV1*, *NKG7*, *MAFA*, *KLF4*, *NFKBIA*
GO:0001568	Blood vessel development	4.67E‐04	*DCN*, *RHOB*, *CXCR4*, *TIGIT*, *EGR3*, *CAV1*, *FMO1*, *KLF4*, *DCLK1*, *NFKBIA*
GO:0043067	Regulation of programmed cell death	5.82E‐04	*HSP90AA1*, *DAPK2*, *RHOB*, *DDIT4*, *PARD6G*, *CXCR4*, *DNAJB1*, *NLRP3*, *TIGIT*, *CAV1*, *MAFA*, *KLF4*, *NFKBIA*
GO:0030334	Regulation of cell migration	7.19E‐04	*DUSP1*, *DAPK2*, *DCN*, *RHOB*, *AMIGO2*, *CXCR4*, *DNAJB1*, *EGR3*, *CAV1*, *FMO1*, *COL1A1*
GO:0006915	Apoptotic process	7.59E‐04	*HSP90AA1*, *DAPK2*, *RHOB*, *TUBB2A*, *DDIT4*, *PARD6G*, *CXCR4*, *DNAJB1*, *NLRP3*, *TIGIT*, *CAV1*, *MAFA*, *KLF4*, *NFKBIA*
GO:0048523	Negative regulation of cellular process	8.44E‐04	*GFI1*, *ADAMTS5*, *RGS1*, *DUSP2*, *DUSP1*, *DCN*, *EREG*, *RHOB*, *TUBB2A*, *DDIT4*, *PARD6G*, *CXCR4*, *PTGS2*, *DNAJB1*, *HSPH1*, *LMNA*, *NLRP3*, *TIGIT*, *CAV1*, *HSPA6*, *FMO1*, *MAFA*, *KLF4*, *NFKBIA*, *COL1A1*
GO:0005584	Collagen type I trimer	9.36E‐04	*FMO1*, *DCLK1*
KEGG:05418	Fluid shear stress and atherosclerosis	1.70E‐03	*CALML5*, *DUSP1*, *NLRP3*, *EGR3*, *CAVIN1*
GO:0033554	Cellular response to stress	2.38E‐03	*CDKN1A*, *GFI1*, *HSP90AA1*, *RHOB*, *TUBB2A*, *PARD6G*, *PTGS2*, *DNAJB1*, *NLRP3*, *EGR3*, *HSPA1L*, *CAV1*, *MAFA*, *NFKBIA*
GO:0001525	Angiogenesis	3.78E‐03	*DCN*, *RHOB*, *CXCR4*, *TIGIT*, *EGR3*, *CAV1*, *KLF4*, *NFKBIA*
GO:0044183	Protein folding chaperone	4.00E‐03	*HSP90AA1*, *PTGS2*, *GATA2*, *HSPA1L*
GO:0043534	Blood vessel endothelial cell migration	4.29E‐03	*CXCR4*, *TIGIT*, *EGR3*, *CAV1*, *KLF4*
GO:0009968	Negative regulation of signal transduction	4.30E‐03	*RGS1*, *DUSP2*, *DUSP1*, *DCN*, *TUBB2A*, *PARD6G*, *CXCR4*, *DNAJB1*, *HSPH1*, *CAV1*, *MAFA*, *NFKBIA*
GO:0008134	Transcription factor binding	5.44E‐03	*DDIT4*, *CXCR4*, *HSPH1*, *NLRP3*, *CAV1*, *NKG7*, *MAFA*, *KLF4*
GO:0002042	Cell migration involved in sprouting angiogenesis	5.84E‐03	*CXCR4*, *TIGIT*, *CAV1*, *KLF4*
GO:0048583	Regulation of response to stimulus	6.14E‐03	*GFI1*, *RGS1*, *HSP90AA1*, *DUSP2*, *DUSP1*, *DAPK2*, *DCN*, *EREG*, *TUBB2A*, *AMIGO2*, *PARD6G*, *CXCR4*, *DNAJB1*, *HSPH1*, *CAV1*, *NKG7*, *FMO1*, *MAFA*, *NFKBIA*, *COL1A1*
GO:0050789	Regulation of biological process	7.07E‐03	*FAAH*, *CDKN1A*, *GFI1*, *PREX2*, *ADAMTS5*, *RGS1*, *HSP90AA1*, *DUSP2*, *DUSP1*, *DAPK2*, *DCN*, *EREG*, *RHOB*, *TUBB2A*, *DDIT4*, *CKS2*, *SIK1*, *AMIGO2*, *PARD6G*, *CXCR4*, *PTGS2*, *DNAJB1*, *HSPH1*, *LMNA*, *NLRP3*, *TIGIT*, *JUN*, *EGR3*, *NOS3*, *CAV1*, *HSPA6*, *NKG7*, *FMO1*, *MAFA*, *KLF4*, *DCLK1*, *NFKBIA*, *COL1A1*
HP:0012888	Abnormal uterine cervix morphology	8.67E‐03	*AMIGO2*, *CXCR4*, *FMO1*
KEGG:04625	C‐type lectin receptor signalling pathway	9.71E‐03	*CALML5*, *HSPH1*, *NLRP3*, *TIGIT*
GO:0009653	Anatomical structure morphogenesis	1.32E‐02	*PREX2*, *ADAMTS5*, *DUSP2*, *DUSP1*, *DCN*, *RHOB*, *CXCR4*, *TIGIT*, *EGR3*, *CAV1*, *HSPA6*, *FMO1*, *MAFA*, *KLF4*, *DCLK1*, *NFKBIA*
GO:0023051	Regulation of signalling	1.33E‐02	*FAAH*, *GFI1*, *PREX2*, *RGS1*, *DUSP2*, *DUSP1*, *DAPK2*, *DCN*, *TUBB2A*, *AMIGO2*, *PARD6G*, *CXCR4*, *DNAJB1*, *HSPH1*, *CAV1*, *FMO1*, *MAFA*, *NFKBIA*
GO:0048731	System development	1.44E‐02	*GFI1*, *PREX2*, *ADAMTS5*, *DCN*, *RHOB*, *DDIT4*, *CKS2*, *AMIGO2*, *CXCR4*, *DNAJB1*, *TIGIT*, *EGR3*, *CAV1*, *HSPA6*, *FMO1*, *MAFA*, *KLF4*, *DCLK1*, *NFKBIA*
GO:1904018	Positive regulation of vasculature development	1.48E‐02	*RHOB*, *AMIGO2*, *CXCR4*, *EGR3*, *CAV1*
GO:0048514	Blood vessel morphogenesis	1.49E‐02	*DCN*, *RHOB*, *CXCR4*, *TIGIT*, *EGR3*, *CAV1*, *KLF4*, *NFKBIA*
GO:1901342	Regulation of vasculature development	1.62E‐02	*DCN, RHOB*, *AMIGO2*, *CXCR4*, *EGR3*, *CAV1*
HP:0005897	Severe generalised osteoporosis	1.65E‐02	*FMO1*, *DCLK1*
HP:0003321	Biconcave flattened vertebrae	1.65E‐02	*FMO1*, *DCLK1*
HP:0005623	Absent ossification of calvaria	1.65E‐02	*FMO1*, *DCLK1*
GO:0044419	Biological process involved in interspecies interaction between organisms	1.92E‐02	*GFI1*, *ADAMTS5*, *RGS1*, *HSP90AA1*, *EREG*, *TUBB2A*, *PARD6G*, *NLRP3*, *EGR3*, *HSPA6*, *NKG7*, *KLF4*
GO:0065007	Biological regulation	1.95E‐02	*FAAH*, *CDKN1A*, *GFI1*, *PREX2*, *ADAMTS5*, *RGS1*, *HSP90AA1*, *DUSP2*, *DUSP1*, *DAPK2*, *DCN*, *EREG*, *RHOB*, *TUBB2A*, *DDIT4*, *CKS2*, *SIK1*, *AMIGO2*, *PARD6G*, *CXCR4*, *PTGS2*, *DNAJB1*, *HSPH1*, *LMNA*, *NLRP3*, *TIGIT*, *JUN*, *EGR3*, *NOS3*, *CAV1*, *HSPA6*, *NKG7*, *FMO1*, *MAFA*, *KLF4*, *DCLK1*, *NFKBIA*, *COL1A1*
GO:0010594	Regulation of endothelial cell migration	2.26E‐02	*DCN*, *RHOB*, *CXCR4*, *EGR3*, *CAV1*
GO:0001228	DNA‐binding transcription activator activity, RNA polymerase II‐specific	2.36E‐02	*CXCR4*, *NLRP3*, *TIGIT*, *NOS3*, *CAV1*, *MAFA*, *KLF4*
HP:0031869	Recurrent joint dislocation	2.37E‐02	*DNAJB1*, *FMO1*, *DCLK1*
GO:0001666	Response to hypoxia	2.52E‐02	*TUBB2A*, *AMIGO2*, *PARD6G*, *DNAJB1*, *MAFA*
GO:0001216	DNA‐binding transcription activator activity	2.60E‐02	*CXCR4*, *NLRP3*, *TIGIT*, *NOS3*, *CAV1*, *MAFA*, *KLF4*
GO:0061629	RNA polymerase II‐specific DNA‐binding transcription factor binding	2.75E‐02	*CXCR4*, *NLRP3*, *CAV1*, *NKG7*, *MAFA*, *KLF4*
GO:0036293	Response to decreased oxygen levels	2.80E‐02	*TUBB2A*, *AMIGO2*, *PARD6G*, *DNAJB1*, *MAFA*
GO:0140662	ATP‐dependent protein folding chaperone	2.95E‐02	*HSP90AA1*, *GATA2*, *HSPA1L*
GO:0034405	Response to fluid shear stress	3.23E‐02	*PARD6G*, *EGR3*, *CAV1*
GO:0004517	Nitric‐oxide synthase activity	3.81E‐02	*HSP90AA1*, *EGR3*
GO:0032502	Developmental process	3.93E‐02	*GFI1*, *PREX2*, *ADAMTS5*, *DUSP2*, *DUSP1*, *DCN*, *EREG*, *RHOB*, *DDIT4*, *CKS2*, *AMIGO2*, *PARD6G*, *CXCR4*, *DNAJB1*, *TIGIT*, *EGR3*, *CAV1*, *HSPA6*, *FMO1*, *MAFA*, *KLF4*, *DCLK1*, *NFKBIA*, *COL1A1*
GO:0009966	Regulation of signal transduction	3.96E‐02	*GFI1*, *RGS1*, *DUSP2*, *DUSP1*, *DAPK2*, *DCN*, *TUBB2A*, *AMIGO2*, *PARD6G*, *CXCR4*, *DNAJB1*, *HSPH1*, *CAV1*, *FMO1*, *MAFA*, *NFKBIA*
GO:0043207	Response to external biotic stimulus	4.59E‐02	*GFI1*, *ADAMTS5*, *RGS1*, *HSP90AA1*, *EREG*, *TUBB2A*, *PARD6G*, *EGR3*, *HSPA6*, *NKG7*, *KLF4*
GO:0070482	Response to oxygen levels	4.75E‐02	*TUBB2A*, *AMIGO2*, *PARD6G*, *DNAJB1*, *MAFA*

*Note:* Padj values shown are g_SCS‐adjusted hypergeometric *p*‐values.

Immediate‐early transcription factors (*FOS*, *JUN*) and heat‐shock proteins (*HSP90AA1*, *HSPA6*, *HSPH1*, *DNAJB1*) dominated the R‐only module, confirming activation of cellular stress and heat‐response programmes. Enrichments included ‘response to stress’ (GO:0006950) and ‘response to hypoxia’ (GO:0001666), indicating induction of protective and repair mechanisms following maximal exertion.

Acute exertion triggered transient immune activation and leukocyte migration, with enrichments in ‘cell locomotion’ (GO:0040011), ‘cell motility’ (GO:0048870), ‘cell migration’ (GO:0016477) and ‘C‐type lectin receptor signalling’ (KEGG:04625). Genes such as *NLRP3*, *CXCR4*, *RHOB* and *EGR3* were strongly up‐regulated, reflecting immune recruitment and vascular communication.

The network showed significant enrichment for ‘vasculature development’ (GO:0001944) and ‘blood vessel morphogenesis’ (GO:0048514). Mechanosensitive regulators (*KLF4*, *CAV1*) and chemotactic mediators (*CXCR4*, *EGR3*) were up‐regulated, while *COL1A1*, *COL1A2* and *DCN* (decorin) were down‐regulated, suggesting transient extracellular matrix (ECM) remodelling.

Enrichment of ‘programmed cell death’ (GO:0012501) and ‘regulation of apoptotic process’ (GO:0042981) identified *DAPK2* and *NFKBIA* as key regulators balancing apoptotic signalling and cytoprotection. Stress‐protective genes (*HSPA6*, *HSPH1*, *HSP90AA1*) and checkpoint mediators (*GADD45*, *DDIT4*) were up‐regulated, consistent with activation of damage‐control networks.

Enrichments for ‘signal transduction’ (GO:0007165), cell communication (GO:0007154) and transcription factor activity (GO:0140297) highlighted the dominance of regulatory hubs. Master transcription factors (*FOS*, *JUN*, *KLF4*, *EGR3*, *MAF*, *GFI1*) and adaptors (*DUSP1*, *RGS1*) were prominently expressed, suggesting complex modulation of signalling pathways.

Several down‐regulated genes (*PTGS2*, *NLRP3*, *KCNJ2*, *LPAR6*, *RXFP4*, *GATA2*) indicated inhibitory control over inflammatory and metabolic signalling (Figure 7B). Their suppression, coupled with ECM down‐regulation, points to transient restraint of excessive inflammation and tissue remodelling following maximal load.

### 3.4 Functional characterisation of the common gene based PPI network across all exertion types

**FIGURE 8:**
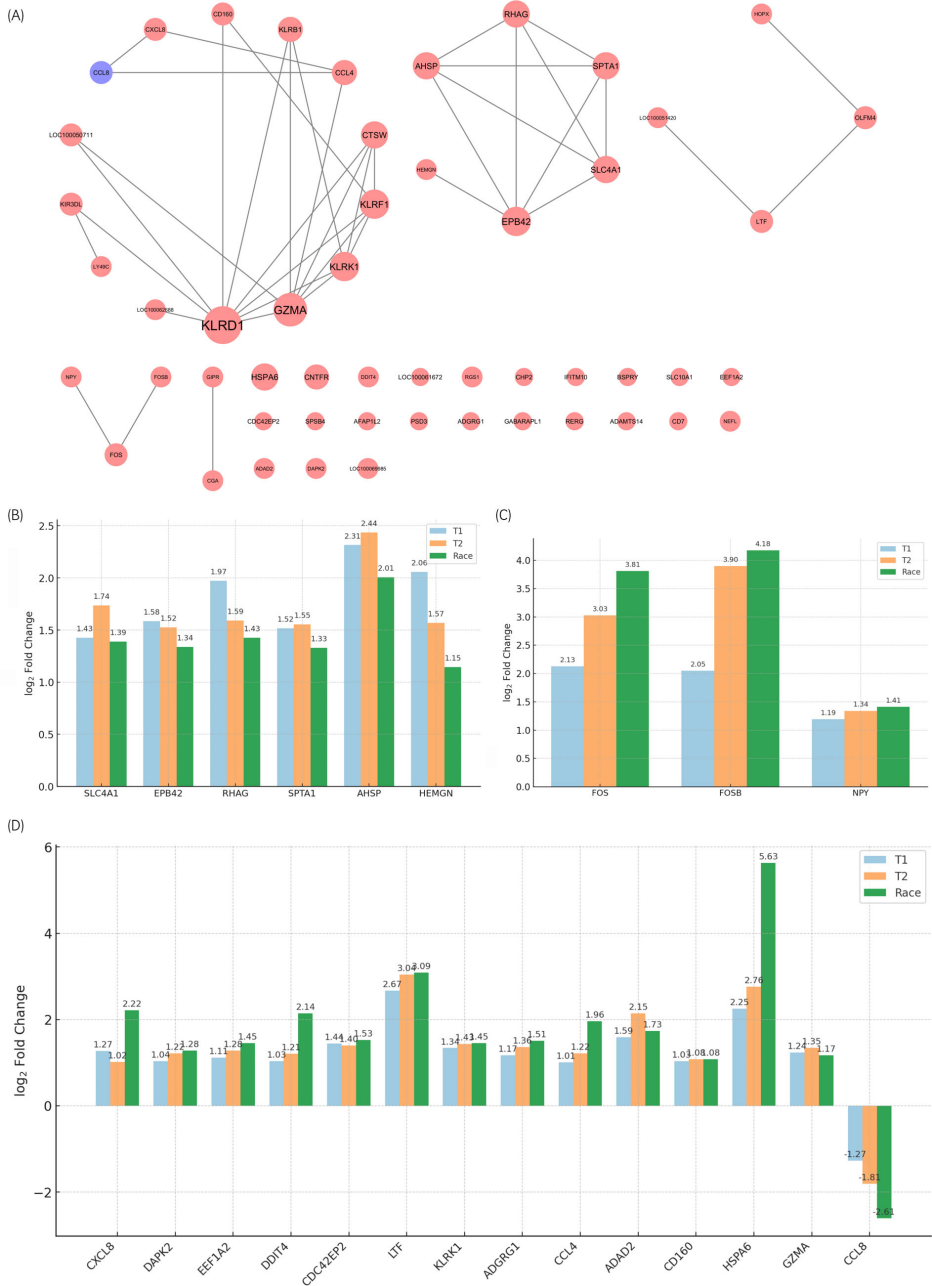
Intersection network and comparative expression profiles across exercise stages. (A) Gene interaction network identified as the intersection of the networks from all exercise conditions (T1, T2 and R). Node colour indicates over (red) or under‐expression (blue). (B) Bar plot of Log_2_ fold change of erythrocyte‐related genes, (C) immediate early and neuroendocrine genes and (D) immune‐related genes across exercise stages across T1, T2 and R conditions.

Across all exertion states, a core PPI network integrating erythroid, immune and stress‐response functions was identified (Figure 8A). The network comprised two large functional modules, three smaller modules (≤4 edges) and several non‐networking genes. It displayed a high degree of interconnectivity between immune and erythrocyte‐associated nodes, with enrichments in haemoglobin binding, MHC class Ib receptor activity, NK cell cytotoxicity and cellular stress response.

The erythroid module, including *SLC4A1*, *EPB42*, *SPTA1*, *HEMGN*, *RHAG* and *AHSP*, was enriched in the terms ‘increased red cell osmotic fragility’ (HP:0005502) and haemoglobin binding (GO:0030492) (Table 8). These genes were consistently up‐regulated across all exertion stages (log_2_FC ≈ +1.3–2.4) (Figure 8B). Additional enrichment included ‘Hyperbilirubinemia’ and ‘Reticulocytosis’.

**TABLE 8:** Terms identified in the functional enrichment of genes shared across all exercise conditions.

Term ID	Term name	*p*‐value	Intersecting genes
HP:0004444	Spherocytosis	1.97E‐07	*SLC4A1*, *HEMGN*, *EPB42*, *LY49C*
HP:0005502	Increased red cell osmotic fragility	2.78E‐06	*SLC4A1*, *HEMGN*, *EPB42*, *LY49C*
HP:0020054	Abnormal erythrocyte physiology	4.01E‐06	*SLC4A1*, *HEMGN*, *EPB42*, *LY49C*
GO:0032394	MHC class Ib receptor activity	8.61E‐06	*SPTA1*, *ADAD2*, *HSPA6*
GO:0170014	Ankyrin‐1 complex	2.51E‐05	*SLC4A1*, *HEMGN*, *LY49C*
HP:0025546	Abnormal mean corpuscular haemoglobin concentration	3.34E‐05	*SLC4A1*, *HEMGN*, *EPB42*, *LY49C*
HP:0005525	Spontaneous haemolytic crises	1.62E‐04	*SLC4A1*, *HEMGN*, *EPB42*
HP:0001923	Reticulocytosis	6.14E‐04	*SLC4A1*, *HEMGN*, *EPB42*, *LY49C*
KEGG:04657	IL‐17 signalling pathway	1.43E‐03	*CXCL8*, *LOC100061672*, *ADGRG1*, *GZMA*
HP:0002904	Hyperbilirubinemia	1.45E‐03	*SLC4A1*, *HEMGN*, *EPB42*, *CGA*, *LY49C*
HP:0033479	Abnormal circulating bilirubin concentration	1.51E‐03	*SLC4A1*, *HEMGN*, *EPB42*, *CGA*, *LY49C*
HP:0004446	Stomatocytosis	1.68E‐03	*SLC4A1*, *EPB42*, *LY49C*
GO:0002376	Immune system process	1.96E‐03	*CXCL8*, *SLC4A1*, *LOC100062688*, *DAPK2*, *LOC100061672*, *EEF1A2*, *DDIT4*, *CDC42EP2*, *LTF*, *HEMGN*, *EPB42*, *SPTA1*, *ADGRG1*, *KLRK1*, *CCL4*, *ADAD2*, *CD160*, *HSPA6*
HP:0004447	Poikilocytosis	1.99E‐03	*SLC4A1*, *HEMGN*, *EPB42*, *LY49C*
HP:0100724	Hypercoagulability	2.09E‐03	*SLC4A1*, *HEMGN*, *EPB42*
GO:0042269	Regulation of natural killer cell‐mediated cytotoxicity	2.37E‐03	*LTF*, *SPTA1*, *ADAD2*, *HSPA6*
HP:0025548	Increased mean corpuscular haemoglobin concentration	3.12E‐03	*SLC4A1*, *HEMGN*, *EPB42*
GO:0002715	Regulation of natural killer cell‐mediated immunity	3.40E‐03	*LTF*, *SPTA1*, *ADAD2*, *HSPA6*
HP:0001978	Extramedullary haematopoiesis	3.74E‐03	*SLC4A1*, *HEMGN*, *EPB42*
HP:0004312	Abnormal reticulocyte morphology	4.12E‐03	*SLC4A1*, *HEMGN*, *EPB42*, *LY49C*
HP:0010995	Abnormal circulating dicarboxylic acid concentration	4.36E‐03	*SLC4A1*, *HEMGN*, *EPB42*, *CGA*, *LY49C*
HP:0001997	Gout	4.44E‐03	*SLC4A1*, *HEMGN*, *EPB42*
HP:0040186	Maculopapular exanthema	4.44E‐03	*SLC4A1*, *HEMGN*, *EPB42*
KEGG:05332	Graft‐versus‐host disease	5.38E‐03	*LOC100050711*, *CCL4*, *HSPA6*
KEGG:04650	Natural killer cell‐mediated cytotoxicity	5.47E‐03	*LOC100050711*, *SPTA1*, *CCL4*, *HSPA6*
HP:0025143	Chills	6.08E‐03	*SLC4A1*, *HEMGN*, *EPB42*
HP:4000054	Exanthem	6.08E‐03	*SLC4A1*, *HEMGN*, *EPB42*
HP:0001723	Restrictive cardiomyopathy	6.08E‐03	*SLC4A1*, *HEMGN*, *EPB42*
KEGG:05144	Malaria	7.98E‐03	*CXCL8*, *LTF*, *SPTA1*
GO:0042267	Natural killer cell‐mediated cytotoxicity	1.10E‐02	*LTF*, *SPTA1*, *ADAD2*, *HSPA6*
HP:0000952	Jaundice	1.77E‐02	*SLC4A1*, *HEMGN*, *EPB42*, *CGA*, *LY49C*
GO:0023024	MHC class I protein complex binding	3.32E‐02	*ADAD2*, *HSPA6*
GO:0001906	Cell killing	4.25E‐02	*CDC42EP2*, *LTF*, *SPTA1*, *ADAD2*, *HSPA6*
GO:0030492	Haemoglobin binding	4.97E‐02	*SLC4A1*, *KLRK1*
GO:0042581	Specific granule	4.98E‐02	*FOS*, *CDC42EP2*

The intersected network also showed significant enrichment in immune‐related pathways such as ‘NK cell‐mediated cytotoxicity’ (KEGG:04650; GO:0042267), Regulation of ‘NK cell‐mediated immunity’ (GO:0002715) and ‘cell killing’ (GO:0001906). Hub genes included *LTF*, *HSPA6*, *SPTA1*, *ADAD2*, *CCL4* and *KLRK1* (Figure 8D).

Further enrichment was observed in ‘IL‐17 signalling’ (KEGG:04657) through *CXCL8*, *GZMA* and *ADGRG1*. Stress‐responsive transcription factors *FOS* and *FOSB* were markedly up‐regulated, particularly during race conditions, while *NPY* exhibited sustained elevation across all exertion stages (Figure 8C).

### 3.5 Breed‐ and sex‐dependent transcriptomic differences

Analysis of sex‐ and breed‐related transcriptional differences using limma‐voom modelling identified several moderate (|Δlog_2_FC| > 1.0) yet non‐significant expression changes across conditions. During the T1, sex‐dependent variation was observed within breeds: *AFAP1L2* showed milder response in Arabian mares compared with stallions (|Δlog_2_FC| = 1.08; FDR = 0.99), while *HSPA6* was up‐regulated to a higher extent in Thoroughbred mares (|Δlog_2_FC| = 1.58; FDR = 0.8) (Table S10 and Figure S1A,B). Breed comparison revealed that *HSPA6* (|Δlog_2_FC| = 1.68; FDR = 0.99) and *LTF* (|Δlog_2_FC| = 1.64; FDR = 0.99) were both more up‐regulated in Arabians than in Thoroughbreds, though neither reached statistical significance after FDR correction (Table S10 and Figure S2A).

At the T2, *HSPA6* responded to the exertion in Thoroughbred to the highest extent, where up‐regulation was higher in stallions (|Δlog_2_FC| = 1.7; FDR = 0.52). No breed‐related differences have been discovered in this condition (Table S11 and Figures S1C,D and S2B).

Following the R, several sex‐related differences reappeared. In Arabians, *HSPA6* up‐regulation was higher in mares (|Δlog_2_FC| = 1.28; FDR = 0.99), while in Thoroughbreds, *FOSB* (Δlog_2_FC = 1.24; FDR = 0.03) showed higher expression in mares and *RGS1* (|Δlog_2_FC| = 1.19; FDR = 0.83) and *KLRD1* (|Δlog_2_FC| = 1.08; FDR = 0.83) were more highly expressed in stallions (Table S12 and Figure S1E,F). In the breed comparison at this stage, *HSPA6* remained moderately higher over‐expressed in Arabians than Thoroughbreds (|Δlog_2_FC| = 1.20; FDR = 0.74) (Table S12 and Figure 1C).

## 4 DISCUSSION

The present study resolves a coherent, multi‐stage programme of haematologic immune activation and cellular stress that unfolds from a first training exposure (T1) through a second bout (T2) and culminates in a race condition, with progressive amplification of immediate‐early transcription factors (*FOS/FOSB/JUN*), inducible chaperones (HSP70‐family, especially *HSPA6*) and NK/cytotoxic modules (*KLRK1/KLRD1/KLRB1*, *PRF1*, *GZMA/GZMB*, *CTSW*, *CCL4*, *CD7/CD160*). Across all exertion types, red‐cell‐linked signals also recur, aligning with known rheological and haemolytic sequelae of equine exercise. Together these data position acute leukocyte activation and erythrocyte turnover as central features of the early transcriptomic response, with repeated training sharpening the cytotoxic/activation axis and the race stimulus superimposing robust proteostasis and trafficking programmes while selectively dampening GPCR and Ig‐like gene families.

### 4.1 Integrated erythroid–immune genes as a common Hallmark across all exertion types

Across all exertion states, we identified a core gene interactome integrating erythroid, immune and stress‐response functions. The erythroid module genes encode cytoskeletal and transport proteins essential for red cell resilience to shear stress. Their consistent up‐regulation across all exercise types indicates reinforcement of the erythrocyte membrane system, not damage, supporting prior physiological findings that training improves RBC deformability and osmotic resistance.^28^, ^29^, ^30^, ^31^ Enrichment of ‘Hyperbilirubinemia’ and ‘Reticulocytosis’ terms further implies elevated turnover and renewal of erythrocytes during exertion.

Moreover, persistent cytotoxic signature suggests a stable immune activation baseline in trained horses, aligning with prior studies reporting post‐exercise immune up‐regulation.^16^, ^20^ The presence of *CXCL8* (IL‐8) and *CCL4* further indicates activation of chemokine signalling, supporting leukocyte trafficking and tissue repair, which has been indicated previously in blood and muscle.^5^, ^32^, ^33^

Additional enrichment in IL‐17 signalling highlights cytokine‐mediated coordination between inflammation and erythropoiesis. In vitro data confirm IL‐17‐mediated induction of *CXCL8* in equine neutrophils, resistant to glucocorticoid suppression, supporting an adaptive inflammatory control mechanism.^34^ Finally, stress‐responsive transcription factors *FOS/FOSB* were markedly up‐regulated, particularly during race conditions, reflecting acute activation of immediate‐early and neuroendocrine pathways. The immediate‐early transcription factor FOS is over‐expressed in peripheral blood mononuclear cells (PBMCs)' response to exercise and has also been shown to rise sharply in equine muscle post‐exertion.^35^ Moreover, *NPY* showed sustained elevation linked to vascular and hormonal regulation.^36^, ^37^

Together, these findings depict a stable multi‐systemic core network that preserves erythrocyte stability, sustains immune vigilance and coordinates cellular stress adaptation across repeated exertion—hallmarks of physiological optimisation in athletic horses.

### 4.2 Acute, first exercise response reflecting rapid immune activation and stress Aignaling (T1)

At T1, the partial separation in global structure and the focused set of DEGs dominated by NK/cytotoxic and immediate‐early signatures mirror prior work in equine blood and muscle showing that a single bout of exercise elicits swift transcriptional waves enriched for AP‐1 factors and immune mediators.^20^, ^35^, ^38^ Notably, *FOS/DDIT4* induction and up‐regulation of *KLRK1/KLRD1*, *PRF1*, *GZMA/GZMB*, *CTSW*, *CCL4*, *CD7/CD160*, and granzyme B (*LOC100052638*) and granzyme H (*LOC100051986/LOC100146914/LOC100146634*) are fully consistent with cellular states captured by single‐cell atlases in horses in which CD3^+^PRF1^+^ cytotoxic lymphocytes (NK/T) organise around granzymes, cathepsin W, killer lectins and chemokines.^38^ Furthermore, Riihimäki et al. (2023) showed γδ‐T and CD8^+^ subsets expressing *KLRK1/KLRD1/KLRB1*, *GZMA/GZMK*, *CTSW*, *CCL5* and adhesion/integrin genes.^39^ It was confirmed that athletic horses have an increased level of cytotoxic CD8+ lymphocytes after exercise.^40^ Additionally, the preferential enrichment of C‐type lectin/NK receptor pathways corroborates these cell‐state inferences and echoes blood‐based racing studies that report post‐exercise up‐regulation of chemokine and leukocyte trafficking modules.^20^, ^23^

Our data similarly highlight a strong induction of innate immune‐related transcripts, including interferon and complement pathways (e.g., omplement C3 [*LOC100060505*], log_2_FC = 1, 8), coupled with selective suppression of immune mediators. Such innate‐immune activation may reflect early stress signalling and immunosurveillance, possibly related to muscle damage and pathogen exposure during exercise. Peripheral blood microarray analyses in Thoroughbreds showed that *IL‐1R2*, *MMP8*, *S100‐A8* and *serum‐amyloid A* (*SAA*) increased 4‐ to 24‐fold within 4 h after maximal exercise and were associated with collagen catabolic and inflammatory processes.^23^ These findings align with our observation of inflammatory chemokines (e.g., *CCL8*, *CCL4*) and suggest that early training induces a neutrophil‐dominated acute phase response.

On the other hand, the predominance of immune‐related pathways contrasts with many previous equine transcriptomic studies, which emphasised metabolic and cell‐cycle pathways during early training. For example, blood transcriptome profiling in Arabian horses across conditioning stages revealed up‐regulation of PI3K–Akt, FoxO, JAK–STAT, cAMP and Notch pathways, while down‐regulated genes were mainly associated with hypertrophic cardiomyopathy.^16^ Similarly, microarray analyses of skeletal muscle in Thoroughbreds detected very few DEGs immediately after exercise but, 4 h later, identified hundreds of genes related to the actin cytoskeleton, MAPK, focal adhesion, insulin and mTOR signalling.^35^

Of special interest is the early induction of *HSPA6* and heat shock 70 kDa protein 1‐like (*LOC111769297*), an HSP70‐family chaperone with robust responsiveness to heat and cellular stress that rises in equine PBMCs within minutes of treadmill work.^24^ The recurrence of *HSPA6* at T1–T2–Race, together with *HSPA1B/HSPH1* in the race condition, fits prior bulk and targeted transcriptomic observations that heat‐shock and proteostasis programmes are prominent features of the equine exercise response.^20^, ^24^, ^41^ Notably, members of the HSP family—including *HSP90AA1*, *HSPA6* and *HSPH1*—are consistently up‐regulated not only in blood but also in muscle, highlighting a conserved cellular stress response critical for protein protection and recovery.^35^ Finally, the appearance of erythrocyte‐related DEGs (e.g., *SLC4A1*, *RHAG*, *SPTA1*, *EPB42*) is biologically plausible in light of the marked exercise‐driven splenic release of red cells in horses and the well‐documented susceptibility of equine RBCs to haemolysis and altered deformability following exertion.^42^, ^43^

These results indicate that acute exercise at the beginning of the training season initiates a broad but disorganised transcriptional response, involving immune surveillance, transient inflammatory suppression and early structural adaptation, consistent with an initial ‘learning phase’ of physiological conditioning.

### 4.3 Amplification and ‘priming’ of the cytotoxic/activation axis following repeated training (T2)

With the second training bout, the transcriptional landscape broadened substantially, reflecting the transition from acute stress responses to long‐term physiological adaptation. The DEG burden expanded markedly (108 up‐regulated and 7 down‐regulated at T1 vs. 360 up‐regulated and 15 down‐regulated at T2), indicating that repeated exertion engages a more complex and integrated response network. The increase in DEGs from T1 to T2 suggests that repeated, moderate exertion does not attenuate the acute transcriptomic reaction but instead elicits a more extensive and functionally integrated response during mid‐season conditioning. These convergent findings are consistent with a training‐induced ‘priming’ effect. Despite differences in sampling design or analytical thresholds, other studies support this trend. Ropka‐Molik et al. reported that the number of DEGs increased across successive training periods, with the largest set observed at the end of the season.^12^, ^16^, ^44^

Repeated training intensified the NK/cytotoxic/immediate‐early gene core identified at earlier stages, suggesting that circulating cytotoxic compartments become ‘primed’ through repeated exposure. FOS and FOSB remained up‐regulated, with higher magnitudes at T2 (log_2_FC 3.02 and 3.9) compared to T1 (2.13 and 2.04), reflecting a sustained transcriptional readiness. This ‘priming’ effect parallels skeletal muscle transcriptomic plasticity described in Thoroughbreds, where training reprogrammes baseline gene expression for faster or stronger responses to subsequent exercise.^44^

Although acute inflammatory responses persisted, they appeared moderated at T2, accompanied by new categories of genes linked to recovery and remodelling. Notably, *HSPA6* (HSP70B′) and *HSPA1B* (HSP70‐1B), key chaperones mediating protein folding and cellular protection, were strongly induced, alongside *AHSP*, *RETREG1*, *HOPX* and *DUSP4*, which support haemoglobin stabilisation, endoplasmic reticulum (ER) quality control, tissue differentiation and MAPK feedback inhibition, respectively. These genes complement cytotoxic mediators (*PRF1*, *GZMA/B*, *KLRD1*) to sustain cellular integrity, controlled inflammation and stress adaptation. Conversely, modest down‐regulation of *CCL8* and structural or GPCR‐related genes (*ARHGAP44*, *OR52B2*, *SALL3*, *UPK3B*) suggests a fine‐tuning of leukocyte trafficking and receptor sensitivity as animals adapt to repeated effort.

#### 4.3.1 Metabolic refinement and adaptive efficiency

Transcriptomic refinement at T2 may also reflect increased metabolic efficiency. Previous studies have shown enhanced mitochondrial and fatty acid metabolism (e.g., *ACADVL*, *MRPS21*) and suppression of growth inhibitors like myostatin (MSTN) during endurance training,^35^ consistent with improved oxidative capacity. Additionally, prior research has shown that long‐term training up‐regulates genes related to energy metabolism, cellular communication and tissue repair. In Arabian horse blood, the transition from early training to peak fitness (T3 in Ropka‐Molik et al. study) was associated with significant enrichment of pathways like PI3K–Akt signalling (cell cycle and survival), cAMP signalling and JAK–STAT signalling (immune modulation).^16^ We observed similar trends at T2: among the significantly up‐regulated transcripts were *IRS3*, an adaptor in the PI3K–Akt insulin/IGF signalling cascade that supports glucose uptake and energy metabolism; *NR4A3*, a nuclear receptor transcription factor known to regulate mitochondrial biogenesis, glucose metabolism and stress resistance in response to exercise; and *DUSP4*, a dual‐specificity phosphatase providing feedback control of MAPK signalling and thereby tempering inflammatory responses while maintaining pro‐survival signalling. These genes were not enriched in response to T1. Thus, the induction of these genes suggests that repeated training primes leukocytes for improved metabolic flexibility, stress tolerance and controlled immune regulation, consistent with the emergence of a trained phenotype.

Importantly, repeated training seems to induce a ‘new normal’ in the immune system. Some immune regulatory genes were down‐regulated with chronic training, which might reflect reduced baseline inflammation and a more efficient immune status. Capomaccio et al. noted that genes regulating immune processes were down‐regulated after intense exercise stress, possibly as a feedback mechanism. Similarly, training status in Thoroughbreds has been shown to alter immune gene expression even at rest—one study observed changes in immune defence genes in trained versus untrained horses.^20^ This suggests that by T2 our horses' immune cells might be preconditioned, exhibiting tempered inflammatory responses and enhanced stress tolerance. Indeed, our data show that heat shock proteins (HSPs) like *HSPA6* (HSP70B′) were moderately up‐regulated in T2 (log2FC ~2.7). Such elevations in molecular chaperones imply that repeated exercise boosts the horse's cellular protein‐folding and stress–resilience machinery, a known adaptation that helps prevent damage from ongoing physical strain.

#### 4.3.2 Growth factor and cytokine signalling modules

Exclusively for T2 gene set, the enrichment of receptor ligand activity driven by *PDGFB*, *VEGFC*, *FGF5*, *FGF18* and *IFNG* reveals coordinated activation of growth and cytokine signalling. *PDGFB* and *VEGFC* promote angiogenesis and endothelial proliferation, supporting capillarisation and nutrient delivery under sustained workload. The exclusive appearance of *VEGFC* in the T2 network points to advanced vascular adaptation, consistent with equine studies showing exercise‐induced *VEGF* up‐regulation.^45^, ^46^ Some studies proposed that understanding *VEGF* dynamics can help optimise training and recovery strategies in horses, potentially enhancing their athletic performance and recovery from injuries.^47^, ^48^ Moreover, *VEGF* gene therapy has been beneficial in treating suspensory desmitis.^48^ This evidence proves that the appearance of *VEGFC* exclusively in the T2 network indicates enhanced training outcomes and recovery as athletes adapt to repeated exercise.

Vascular endothelial growth factor and insulin receptor (*INSR*) exclusive in response to T2 were also identified as exercise responsive genes in equine muscles. This suggests that PBMCs can reflect key elements of the muscle's response to training at some point.^44^

*FGF5* and *FGF18* pair adds a dimension of tissue remodelling and muscle maintenance, suggesting sustained support of myogenic and connective processes. One study on horses indicated that *FGF2*, another member of the FGF family, significantly increased satellite cell proliferation, which is crucial for muscle growth and repair.^49^ This suggests that FGFs, in general, play a role in muscle adaptation to exercise. Conversely, Ropka‐Molik et al. report down‐regulation of FGF during repeated exercise.^44^

#### 4.3.3 JAK–STAT signalling as an adaptive hub

The concurrent presence of IL‐13 and IFN‐γ suggests that repeated training fine‐tunes inflammation through JAK–STAT‐mediated feedback, facilitating recovery and homeostasis.^50^ This pathway was likewise enriched in season‐end transcriptomes of trained horses,^16^ and recognised as a key mediator of adaptive training responses in humans.^51^ In racehorses, higher concentration of IL‐13 after race specifically in trained horses has been reported, which was not the case for the untrained group.^2^

The inclusion of *IL22RA1*, a receptor for IL‐22, points to anti‐inflammatory and antioxidative functions supporting post‐exercise recovery.^52^ Given its additional role in hepatic lipid metabolism and energy balance,^53^ *IL22RA1* expression likely contributes to the regulation of lipid utilisation during prolonged effort—a crucial adaptation for horses, which increasingly rely on lipid oxidation for sustained performance.^31^ Finally, the co‐expression of *IFNG* and *IL13* reflects the coexistence of pro‐ and anti‐inflammatory signalling, characteristic of immune equilibrium in well‐conditioned horses.^40^

#### 4.3.4 Neuroendocrine and neuromuscular remodelling

The gene set exclusive for T2 also revealed significant neuroendocrine and reproductive signalling enrichment, including *GNRH1*, *ZFPM2*, *PDGFB* and *HFM1*—genes associated with gonadotropin regulation, energy balance and vascular‐endocrine crosstalk.^54^, ^55^, ^56^ Concurrent urocortin (UCN) up‐regulation supports HPA‐axis engagement and stress adaptation, consistent with its cardioprotective and anti‐stress roles.^57^, ^58^, ^59^

*ZFPM2*, a GATA co‐regulator, contributes to vascular and haematopoietic development, suggesting vascular‐endocrine interplay.^60^, ^61^, ^62^

Meanwhile, the over‐expression of *ADD2*, *SNAP25* and *MPP2* is a novel finding in horses and implies cytoskeletal reinforcement and neuromuscular remodelling. *ADD2* stabilises the actin–spectrin network,^63^, ^64^ *SNAP25* modulates vesicle fusion and synaptic plasticity,^65^, ^66^ and MPP2 anchors postsynaptic signalling complexes.^67^, ^68^

Together, these genes indicate coordinated endocrine, vascular and neuromuscular adaptation sustaining long‐term performance. Additional factors such as *MPC1L* (mitochondrial pyruvate carrier),^69^ and meiotic regulators *HFM1* and *MEIOB* may play noncanonical metabolic or repair roles under exercise stress, warranting their further validation in equine models.^70^

### 4.4 Race‐specific transcriptional signatures reflect acute stress, immune activation and vascular remodelling

The race‐specific transcriptome represents the most extreme molecular state observed in this study, defining a distinct phenotype of acute physiological overload and adaptive protection. This R‐exclusive gene set, absent from the training conditions, reveals how maximal exertion reconfigures transcriptional programmes to prioritise survival, repair and transient immune mobilisation.

#### 4.4.1 Immediate stress response and cytoprotection

Immediate‐early transcription factors (*FOS*, *JUN*) and heat‐shock proteins (*HSP90AA1*, *HSPA6*, *HSPH1*, *DNAJB1*) dominated the R‐only module, confirming a strong cellular stress and heat response consistent with prior equine studies.^23^, ^35^, ^71^ *HSPA6* showed an over fivefold induction post‐race, more than twice that seen at T1, indicating an intense heat shock response triggered by the extreme metabolic load. The coordinated up‐regulation of HSP70‐family members mirrors findings from endurance and sprint horses, confirming conserved activation of protein‐folding and recovery pathways.^23^, ^71^

These findings indicate a robust heat shock response, which is expected because racing can lead to significant heat production, metabolic acidosis and oxidative stress in tissues. The induction of HSPs helps prevent protein denaturation and aids in post‐exercise recovery and has been similarly observed in other studies of intense exercise. In a recent transcriptomic study of Yili horses running a 5000 m race, *HSP90AA1*, *HSPA4* and other HSPs emerged as central hub genes in the blood gene network post‐race, emphasising that chaperone‐mediated protein folding is a key response to acute exercise stress.^32^

Complementing this heat‐shock response, *GADD45* and *DDIT4* (REDD1) were strongly induced, signifying activation of DNA repair and inhibition of mTOR signalling, respectively. *DDIT4* is a known inhibitor of mTOR signalling induced under stress (e.g., low oxygen or energy). It's up‐regulation suggests the horses' cells were responding to the metabolic strain of sprinting by temporarily suppressing growth pathways to conserve energy. This aligns with Capomaccio et al.'s finding of a ‘transient decrease in global protein synthesis’ after strenuous exercise—a protective measure in stress conditions.^20^ Additionally, JUN (c‐Jun, part of AP‐1) was significantly up‐regulated in race samples, indicating activation of stress‐activated protein kinase pathways (like JNK/MAPK). These pathways can drive both inflammation and adaptive gene expression.

#### 4.4.2 Immune activation, migration and feedback regulation

Racing elicited a rapid immune activation marked by up‐regulation of *CXCR4*, *KLRD1*, *CLEC5A*, *HAVCR2* and *CCL4*, which coordinate cytotoxic cell activity and chemokine‐mediated leukocyte redistribution. These findings parallel large‐scale blood studies in endurance horses showing that immediate‐early and immune signalling pathways dominate the post‐ride landscape, where miRNA/mRNA networks converge on stress and inflammatory control.^20^, ^72^ *CXCR4* up‐regulation, in particular, recapitulates breed‐ and intensity‐dependent increases reported by Khummuang et al., underscoring its central role in leukocyte trafficking and vascular‐endothelial communication during recovery. Notably, we did not notice any differences in race response of these genes between Arabian and Thoroughbred.^24^

At the same time, selective down‐regulation of certain immune markers (*IL18*, *PTGS2*) and inflammasome components (*NLRP3*) indicates feedback suppression of excessive inflammation. This dual pattern—the coexistence of pro‐ and anti‐inflammatory regulation—reflects a dynamic immunological balance observed in both equine and human athletes, in which innate immune functions become transiently enhanced while adaptive or pro‐inflammatory responses are temporarily moderated. Such modulation likely prevents prolonged tissue damage while facilitating efficient tissue clearance and repair. The specific down‐regulation of NLRP3 is notable: equine PBMCs harbour a functional NLRP3 inflammasome,^73^ and suppression here may reflect negative feedback on IL‐1β‐linked inflammation once acute demands shift toward resolution, as also suggested in equine airway macrophage work mapping interferon‐ and inflammation‐biased states.^39^, ^74^

In parallel, the race down‐set is enriched for class A rhodopsin‐like GPCRs and Ig‐superfamily glycoproteins, a pattern suggestive of transient attenuation of broad environmental sensing and intercellular adhesion programmes. Similar reductions in olfactory/chemosensory GPCR transcript abundance have been reported in blood after strenuous exercise in other mammalian contexts and in equine studies where immune and signaling tone is rebalanced after competition.^32^, ^75^

#### 4.4.3 Vascular remodelling and structural turnover

Acute haemodynamic and mechanical stress during racing activates vascular and connective‐tissue remodelling processes. The enrichment for vasculature development and blood vessel morphogenesis, with mechanosensitive regulators *KLF4*, *CAV1* and *EGR3* coordinating endothelial responses to shear stress. These signals suggest rapid angiogenic priming and endothelial remodelling akin to vascular responses reported in equine skeletal muscle after intense training.^44^, ^68^

In contrast, *COL1A1*, *COL1A2* and *DCN* (decorin) were down‐regulated, reflecting temporary ECM disassembly and connective‐tissue stress. Decorin, known as an exercise‐induced myokine that promotes muscle hypertrophy by inhibiting MSTN,^76^ may undergo regulated suppression during peak exertion to permit transient remodelling. This combination of angiogenic signalling and ECM reorganisation exemplifies a controlled structural adaptation that supports tissue perfusion and elasticity without promoting fibrosis.

#### 4.4.4 Apoptosis, survival and homeostatic balance

The race transcriptome revealed tight regulation of cell fate through simultaneous activation of pro‐survival and pro‐apoptotic factors. Enrichment of programmed cell death pathways highlighted *DAPK2* (a pro‐apoptotic kinase) and *NFKBIA* (IκBα) as key regulators mediating the balance between damage clearance and cytoprotection. The co‐expression of these genes, alongside chaperones and checkpoint regulators (HSPs, *GADD45*, *DDIT4*), points to a self‐limiting apoptosis‐repair cycle that enables muscle and immune tissues to recover without chronic inflammatory activation.^14^ Negative feedback adaptors (*DUSP1*, *RGS1*) further modulate MAPK and GPCR signalling, ensuring the stress response is intense but transient, preventing systemic overactivation.

#### 4.4.5 Transcriptomic expansion and genomic plasticity

A striking feature of the race transcriptome was broadening of transcriptomic diversity, especially the emergence of uncharacterised and non‐coding RNA species. In our all datasets, a substantial proportion of differentially expressed transcripts are uncharacterised locus (LOC) loci, however, in the Race condition their constitute the highest proportion (T1: 1983 sig genes; 339 LOCs [48 up, 4 down with |log_2_FC| ≥ 1]; T2: 3700 sig; 721 LOCs [182 up, 10 down with |log_2_FC| ≥ 1]; Race: 4358 sig; 1021 LOCs [153 up, 104 down with |log_2_FC| ≥ 1]). DAVID collapses many of these LOCs into domain‐based categories (e.g., rhodopsin‐like GPCR and Ig‐like folds), yielding the observed down‐regulated clusters. Thus, rather than inferring a surge of non‐coding RNA as reported in some race/endurance studies, our data support a LOC‐heavy, domain‐enriched suppression of selected receptor/Ig‐like modules alongside strong up‐regulation of innate cytotoxic/chemokine genes. Under the extreme stress of racing, the horses' cells not only changed expression of known genes but also activated novel transcripts. In the endurance race study, researchers reported a significant increase in reads mapping to intergenic and intronic regions in post‐race samples, with many transcripts not annotated in the reference genome.^20^ They found elevated expression of transposable element sequences (LINE1, LINE2) and antisense RNAs under stress. This suggests that severe physiological stress can trigger widespread genomic ‘noise’ or possibly regulatory RNAs that are normally silent. Similarly, the 5000 m race study on Yili horses identified 4375 lincRNAs differentially expressed (almost all up‐regulated) and 68 circRNAs (mostly up) alongside the mRNA changes.^77^ The lncRNAs were enriched in neuroendocrine signalling pathways, hinting that non‐coding RNAs might modulate the stress response by affecting neuronal and hormonal communication. In our race samples, this phenomenon likely occurred as well—the data showed a broader variance in transcripts after racing, indicating the transcriptomic landscape becomes more complex and dysregulated under maximal stress.^20^ Such activation of normally quiet genomic regions could be a regulatory mechanism (e.g., generating antisense transcripts to modulate gene expression) or a consequence of cellular stress and genomic instability caused by the race. In either case, it underscores how racing pushes the horse's transcriptomic response to a different level, beyond the scope of changes seen with controlled training.

### 4.5 Limited breed‐ and sex‐specific transcriptional divergence

A small number of studies have addressed sex‐specific transcriptomic variation in horses (for example in the Kazakh horse, comparing stallions vs. mares) but without coupling this to an exercise challenge or training adaptation context.^78^ Likewise, studies examining exercise‐induced transcriptomic changes in other breeds rarely stratify by sex or include multiple breed groups. Nonetheless, emerging evidence indicates that molecular responses to exercise can differ between sexes. Female horses tend to up‐regulate genes associated with protein synthesis and muscle recovery, whereas geldings show greater expression of inflammatory and stress‐related genes.^19^ Collectively, these observations suggest potential sex‐dependent regulatory mechanisms in exercise adaptation, yet comprehensive comparative analyses remain scarce. However, in our study, only one gelding was examined. On the other hand, previous studies indicate that horses do not exhibit significant sex‐related differences in muscle structure or physiology, including the composition of carnosine content or histochemical profiles of individual muscle fibres.^79^, ^80^ The variability in muscle‐fibre composition reported in the literature is driven primarily by training level rather than by sex.^81^

Importantly, timepoint‐specific heterogeneity is likely to contribute to the apparent breed divergence at T1. As the earliest stage of the season, T1 may capture a less ‘trained’ and more variable physiological state, in which horses differ in recent workload, acclimation and individual stress responsiveness. Consequently, exercise‐induced transcriptional changes at T1 are expected to show greater inter‐individual dispersion. In contrast, after several weeks of conditioning (T2), the cohort is more likely to have converged toward a comparatively stable trained phenotype, yielding more consistent transcriptional response profiles across breeds. Therefore, any breed‐associated differences observed at T1 (including patterns involving stress‐responsive genes such as HSPA6) should be interpreted cautiously as exploratory and hypothesis‐generating and will require confirmation in a larger, balanced cohort explicitly powered to test breed‐by‐exercise interactions. The absence of statistically significant breed‐ or sex‐specific expression shifts may reflect limited statistical power and unbalanced sampling, notably the small number of Thoroughbred mares (*n* ≈ 3), which inflated variance and reduced sensitivity of interaction terms. The focus on PBMC‐derived RNA rather than skeletal muscle, where exercise responses are pronounced may also have constrained the detectable signal.^14^, ^82^ However, across the full cohort, *HSPA6* is consistently identified as an exercise‐responsive stress gene, supporting its interpretation as a marker of acute cellular heat‐shock response that is most pronounced at early training (T1) and after racing.^24^

As a result, although numerous studies describe how gene expression shifts with exercise within specific breeds and cohorts, we still lack robust data on whether and to what extent, the magnitude, direction or molecular pathways of response differ between breeds or sexes. Consequently, the molecular signatures of exercise adaptation in horses remain broadly characterised rather than stratified by these key biological variables.

### 4.6 Future directions and clinical applications

The molecular signatures identified here provide a proof of concept that whole‐blood RNA can serve as a window into training adaptation and stress physiology in racehorses. Several robust markers of stress and remodelling, such as *FOS/FOSB*, *HSPA6/HSPA1B*, *PRF1/GZMB* and *NR4A3/IRS3/DUSP4*, emerged across different contexts, highlighting candidate transcripts that could be validated as biomarkers of training load and recovery. Validation should be pursued in independent cohorts with targeted qPCR or digital PCR, stratified by breed, sex and training intensity and ideally linked to clinical indicators of fitness, performance and overtraining risk.^83^

Future studies should move beyond single‐time point contrasts. Longitudinal transcriptomic profiling that follows horses across baseline, acute training, chronic conditioning and recovery would help establish individual thresholds of physiological resilience and enable earlier detection of maladaptive responses. To systematise interpretation, we propose composite functional scores derived from targeted gene panels. The Cytotoxic Activation Score (e.g., *KLRD1*, *PRF1*, *GZMB*) could serve as an indicator of transient cytotoxic bursts after exertion, where a slower return to baseline might point to physiological overload. The Heat Shock/Proteostasis Score (e.g., *HSPA6*, *HSPA1B*, *HSPH1*) may show only modest increases during training but a pronounced peak during racing; if elevated at baseline or slow to normalise, it might signal early overreaching. The Regulatory/Adaptation Score (e.g., *NR4A3*, *CXCR4*, *DUSP1*) could reflect the fine‐tuning of inflammatory pathways, with moderate rises perhaps indicating beneficial adaptation. Likewise, the Erythrocyte Turnover/Fragility Score (e.g., *SLC4A1*, *RHAG*, *EPB42*) might highlight exercise‐induced haemolysis or oxidative stress, with persistently high values potentially revealing vulnerability to red‐cell fragility. To explore these dynamics, a 6‐gene screening panel (*HSPA6*, *FOS*, *KLRD1*, *PRF1*, *CXCR4*, *NR4A3*, with *NLRP3* as a down‐regulation marker) may provide a rapid overview, while a 12‐gene panel could offer greater resolution of overload states and a broader 20‐gene panel might allow construction of the functional scores.

Technically, cell‐type deconvolution or single‐cell RNA‐seq could attribute transcriptional programmes to specific leukocyte subsets, distinguishing, for example, NK‐cell activation from monocyte‐derived stress responses. Orthogonal assays—such as plasma cytokines/chemokines, haemolysis markers and immune phenotyping—would strengthen causal inference.^42^, ^84^ Finally, a systems‐level approach integrating transcriptomics with microbiome and metabolome profiling, as shown in endurance cohorts,^74^ could reveal upstream modulators (e.g., SCFAs, PPARγ ligands) that fine‐tune the observed blood transcriptional dynamics. Together, these directions point toward a future in which minimally invasive blood‐based transcriptomics informs individualised training and welfare management in equine athletes.

### 4.7 Study limitations

This study focused exclusively on peripheral blood RNA, which, although practical and minimally invasive, cannot fully capture tissue‐specific events in skeletal muscle, heart or liver, where many training adaptations occur. Nonetheless, blood provides a valuable systemic readout of immune, stress and metabolic regulation and is uniquely suited for serial monitoring in performance horses where invasive biopsies are impractical.

Several interpretive caveats warrant emphasis. The strongest case–control divergence was observed in Race, whereas T2 showed higher within‐group dispersion—this may reflect biological heterogeneity in training adaptation, but could also arise from unmodeled covariates (e.g., circadian sampling effects, degree of haemolysis, subtle breed/sex differences). Adjusting for such covariates and integrating cell fraction estimates would refine resolution.

A further limitation is that we sampled at three discrete timepoints. Rapidly resolving or delayed transcriptional responses may have been missed, although our design successfully captured acute early responses (T1), adaptive remodelling signatures (T2) and race‐induced divergence (R). Importantly, this design mirrors real‐world veterinary practice, where routine pre‐ and post‐exercise blood draws are feasible and thus maintains clinical translatability.

Finally, we observed a large fraction of significant LOC genes, especially at Race. Current annotations classify these as ‘uncharacterised’, complicating biological interpretation. Future use of updated EquCab4 annotation, long‐read RNA‐seq and functional studies will be required to resolve whether these LOCs represent novel protein‐coding genes, lncRNAs or pseudogenes.

Despite these limitations, the study provides a robust and clinically relevant first map of how blood transcriptomes shift across different exercise contexts in competitive horses.

## 5 CONCLUSIONS

In conclusion, the differences in transcriptomic responses at T1, T2 and Race illustrate the trajectory of the equine athlete's body from initial challenge, through adaptation, to extreme performance. Early exercise triggers a wave of stress and immune signals; with repeated training, the horse's transcriptome ‘learns’ and shifts toward a protective, efficient mode; and an intense race elicits both the maximal activation of those learned defenses and some entirely new molecular events.

These findings demonstrate the potential of transcriptomic profiling to identify molecular biomarkers of training load, fatigue and recovery. Genes linked to immune activation and stress may serve as early indicators of adaptation or overload. The broader and more intense transcriptomic shifts observed after racing, marked by immune activation and stress pathway up‐regulation, suggest that recovery demands following competition may exceed those required after standard training sessions. Despite limitations, this approach offers a minimally invasive and informative tool to monitor systemic responses to exercise. In the future, these insights could support precision training strategies and contribute to the development of biomarkers for performance optimization and overtraining prevention in equine athletes.

## FUNDING INFORMATION

This research was funded by the National Science Centre, Poland, No. 2021/41/B/NZ7/03548 (O.W.P.) and the publication was (co)financed by the Science Development Fund of the Warsaw University of Life Sciences—SGGW.

## CONFLICT OF INTEREST STATEMENT

The authors declare that the research was conducted in the absence of any commercial or financial relationships that could be construed as a potential conflict of interest.

## AUTHOR CONTRIBUTIONS

**Izabela Dąbrowska:** Conceptualization; investigation; writing – original draft; methodology; visualization; writing – review and editing; software; formal analysis; data curation. **Jowita Grzędzicka‐Agko:** Investigation; writing – original draft; writing – review and editing. **Paula Kiełbik:** Investigation; writing – review and editing. **Michał Trela:** Investigation; writing – review and editing. **Olga Witkowska‐Piłaszewicz:** Conceptualization; investigation; funding acquisition; writing – original draft; writing – review and editing; formal analysis; supervision; resources; project administration.

## DATA INTEGRITY STATEMENT

Izabela Dąbrowska had full access to all the data in the study and takes responsibility for the integrity of the data and the accuracy of data analysis.

## ETHICAL ANIMAL RESEARCH

All blood sampling procedures were conducted as part of routine health assessments and exercise testing. Following the European Directive 2010/63/EU and Polish regulations on animal experimentation, ethical approval was not required, as these procedures are classified as non‐experimental clinical veterinary practices and are therefore exempt from the directive.

## INFORMED CONSENT

Explicit informed consent for use of clinical data and biological material (blood samples) for research purposes was obtained from the horses' trainer.

## Supporting information


**Figure S1:** Comparison of exercise‐induced gene expression responses between mares and stallions within Arabian and Thoroughbred horses. Scatter plots show log_2_ fold changes (log_2_FC) for the top 30 DEGs identified in the DESeq2 analysis at each condition. For each gene, log_2_FC values in mares (*x*‐axis) are plotted against the corresponding values in stallions (*y*‐axis). (A, C, E) Thoroughbred horses at initial training—T1, mid‐season training—T2 and races—R, respectively; (B, D, F) Arabian horses at the same time points. Each point corresponds to one gene (labelled by gene symbol); the dashed diagonal line indicates identical responses (*y* = *x*) between sexes, with deviations reflecting sex‐specific differences in the transcriptional response within a given breed.

**Figure S2:** Comparison of exercise‐induced gene expression responses between Arabian and Thoroughbred horses. Scatter plots show log_2_ fold changes (log_2_FC) for the top 30 DEGs at each condition. For each gene, log_2_FC values in Arabians (*x*‐axis) are plotted against the corresponding values in Thoroughbreds (*y*‐axis). Panels represent time points initial training—T1 (A), mid‐season training—T2 (B) and races—R (C). Each point corresponds to one gene (labelled by gene symbol); the dashed diagonal line indicates identical responses (*y* = *x*) between breeds, with deviations reflecting breed‐specific differences in the magnitude of the transcriptional response.

**Table S1:** Summary of sample distribution by breed, sex and condition across all time points (T1, T2, R).

**Table S2:** Raw count matrices metadatas.

**Table S3:** Differentially expressed genes at T1, including baseMean, log2FoldChange, lfcSE (standard error of log2 fold change), stat (test statistic), *p*‐value and adjusted *p*‐value (padj).

**Table S4:** Differentially expressed genes at T2, including baseMean, log2FoldChange, lfcSE (standard error of log2 fold change), stat (test statistic), *p*‐value and adjusted *p*‐value (padj).

**Table S5:** Differentially expressed genes at race, including baseMean, log2FoldChange, lfcSE (standard error of log2 fold change), stat (test statistic), *p*‐value and adjusted *p*‐value (padj).

**Table S6:** Sample identifiers metadata and annotations distinguishing control and case groups for Training Session 1 (T1).

**Table S7:** Sample identifiers metadata and annotations distinguishing control and case groups for Training Session 2 (T2).

**Table S8:** Sample identifiers metadata and annotations distinguishing control and case groups for race.

**Table S9:** Summary of Functional annotation clustering of differentially expressed genes using the DAVID platform.

**Table S10:** Summary of statistical model results for the T1 condition. Summary of model results fitted for the top 30 genes identified by DESeq2 analysis, with additional within‐breed sex comparisons (mares vs. stallions) and a combined breed comparison to identify race‐specific transcriptomic responses. Reported statistics include log_2_ fold changes (log_2_FC), moderated *t*‐values, raw *p*‐values and Benjamini–Hochberg adjusted *p*‐values (FDR).

**Table S11:** Summary of statistical model results for the T2 condition. Summary of model results fitted for the top 30 genes identified by DESeq2 analysis, with additional within‐breed sex comparisons (mares vs. stallions) and a combined breed comparison to identify race‐specific transcriptomic responses. Reported statistics include log_2_ fold changes (log_2_FC), moderated *t*‐values, raw *p*‐values and Benjamini–Hochberg adjusted *p*‐values (FDR).

**Table S12:** Summary of statistical model results for the race condition. Summary of model results fitted for the top 30 genes identified by DESeq2 analysis, with additional within‐breed sex comparisons (mares vs. stallions) and a combined breed comparison to identify race‐specific transcriptomic responses. Reported statistics include log_2_ fold changes (log_2_FC), moderated *t*‐values, raw *p*‐values and Benjamini–Hochberg adjusted *p*‐values (FDR).

## Data Availability

The data that support the findings of this study are openly available in RepOD at https://doi.org/10.18150/NSE01R.
