# Supplementary material for: Transcriptomic signatures reveal systemic adaptations and immune modulation in response to training and competitive racing in horses
Source: Equine Vet J. 2026 Mar 7;58(5):1413–44. doi: 10.1002/evj.70154 (PMC13447954; doi:10.1002/evj.70154)
Supplement: Supplementary file 1 [file EVJ-58-1413-s010.pdf]

Equine Veterinary Journal BEVA

Table S1: Summary of sample distribution by breed, sex, and condition across all time points (T1, T2, R).

Condition	Group	Arabian Mares	Arabian Stallions	Thoroughbred Mares	Thoroughbred Stallions	Unique Horses
**T1**	Case	7	16	3	7	32
	Control	8	16	3	6	33
**T2**	Case	9	18	5	6	37
	Control	9	19	6	5	38
**R**	Case	7	15	4	6	32
	Control	7	15	4	6	32
