# Supplementary material for: Transcriptomic signatures reveal systemic adaptations and immune modulation in response to training and competitive racing in horses
Source: Equine Vet J. 2026 Mar 7;58(5):1413–44. doi: 10.1002/evj.70154 (PMC13447954; doi:10.1002/evj.70154)

Figure S2: Comparison of exercise-induced gene expression responses between Arabian and Thoroughbred horses. Scatter plots show $\log_2$ fold changes ($\log_2$FC) for the top 30 DEGs at each condition. For each gene, $\log_2$FC values in Arabians (x-axis) are plotted against the corresponding values in Thoroughbreds (y-axis). Panels represent time points initial training - T1 (A), mid-season training - T2 (B) and races - R (C). Each point corresponds to one gene (labelled by gene symbol); the dashed diagonal line indicates identical responses (y = x) between breeds, with deviations reflecting breed-specific differences in the magnitude of the transcriptional response.

Equine Veterinary Journal BEVA

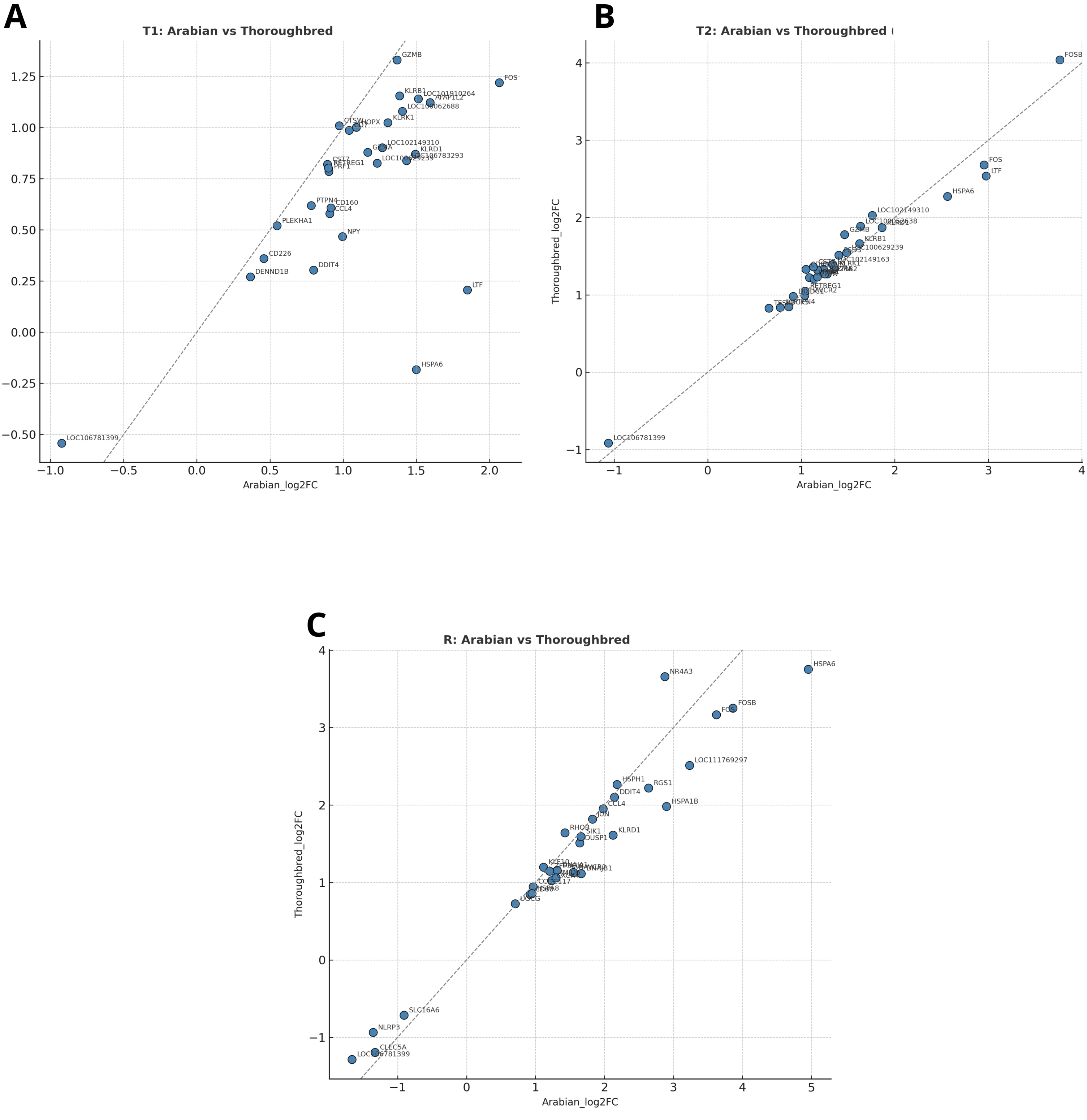

Supplement: Supplementary file 2 [file EVJ-58-1413-s013.pdf]
