# Supplementary material for: Transcriptomic signatures reveal systemic adaptations and immune modulation in response to training and competitive racing in horses
Source: Equine Vet J. 2026 Mar 7;58(5):1413–44. doi: 10.1002/evj.70154 (PMC13447954; doi:10.1002/evj.70154)
Supplement: Supplementary file 3 [file EVJ-58-1413-s014.pdf]

Equine Veterinary Journal BEVA

Figure S1: Comparison of exercise-induced gene expression responses between mares and stallions within Arabian and Thoroughbred horses. Scatter plots show $\log_2$ fold changes ($\log_2$FC) for the top 30 DEGs identified in the DESeq2 analysis at each condition. For each gene, $\log_2$FC values in mares (x-axis) are plotted against the corresponding values in stallions (y-axis). Panels A, C and E show Thoroughbred horses at initial training - T1, mid-season training - T2 and races - R, respectively; panels B, D and F show Arabian horses at the same time points. Each point corresponds to one gene (labelled by gene symbol); the dashed diagonal line indicates identical responses (y = x) between sexes, with deviations reflecting sex-specific differences in the transcriptional response within a given breed.

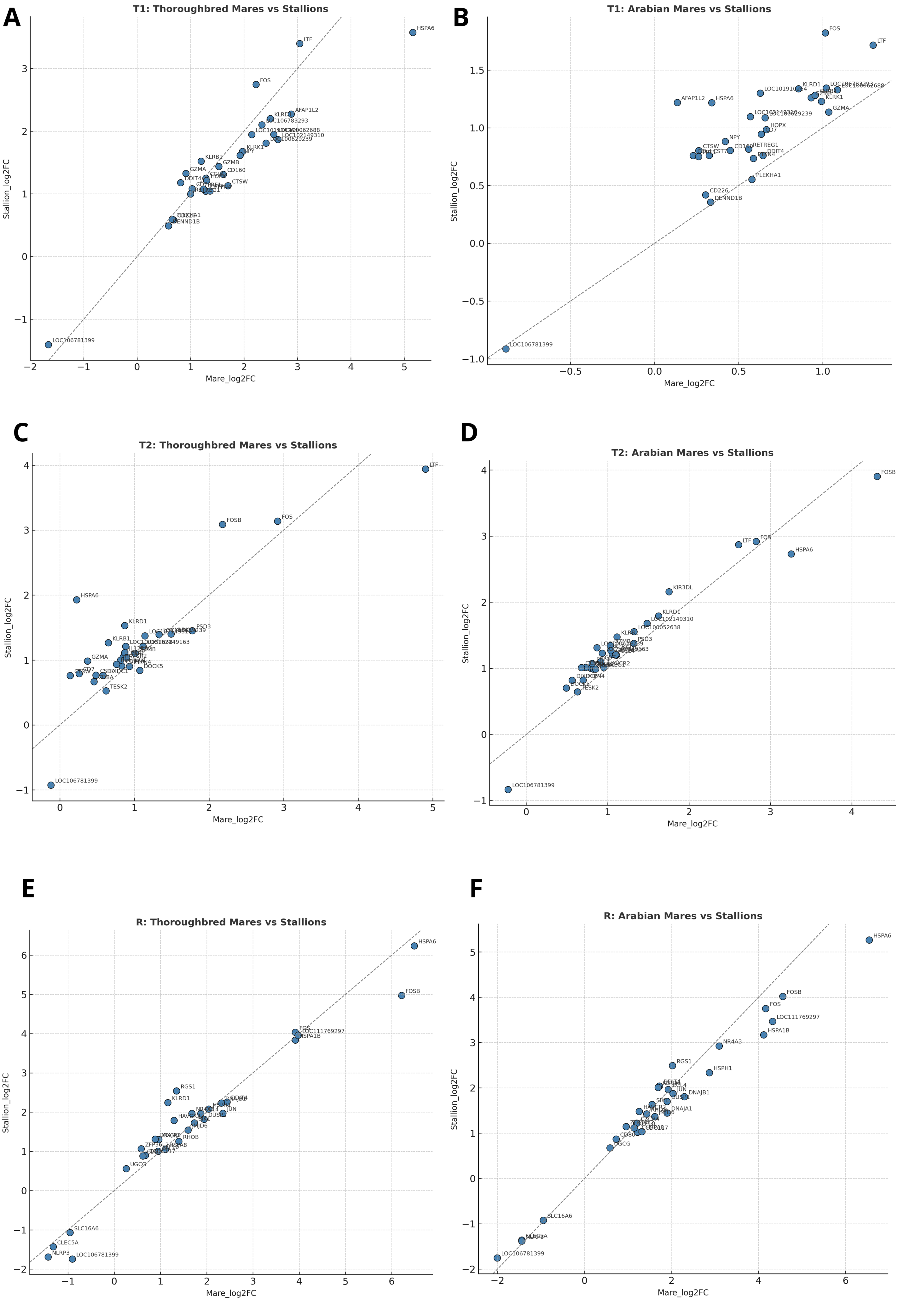
A
T1: Thoroughbred Mares vs Stallions
Stallion_log2FC
Mare_log2FC
HSPA6
LTF
FOS
AFAP1L2
KLRD1
LOC106783293
KLRK1
NPY
KLRB1
GZMB
GZMA
CD160
DDIT4
CTSW
PLEKHA1
DENND1B
LOC106781399
B
T1: Arabian Mares vs Stallions
Stallion_log2FC
Mare_log2FC
FOS
LTF
KLRD1
AFAP1L2
HSPA6
KLRK1
GZMA
HOPX
NPY
CTSW
CD160
RETREG1
DDIT4
PLEKHA1
CD226
DENND1B
LOC106781399
C
T2: Thoroughbred Mares vs Stallions
Stallion_log2FC
Mare_log2FC
LTF
FOSB
FOS
HSPA6
KLRD1
PSD3
KLRB1
GZMA
CD7
DOCK5
TESK2
LOC106781399
D
T2: Arabian Mares vs Stallions
Stallion_log2FC
Mare_log2FC
FOSB
LTF
FOS
HSPA6
KIR3DL
KLRD1
LOC102149310
LOC100052638
PSD3
TESK2
LOC106781399
E
R: Thoroughbred Mares vs Stallions
Stallion_log2FC
Mare_log2FC
HSPA6
FOSB
FOS
LOC111769297
HSPA1B
RGS1
KLRD1
JUN
RHOB
UGCG
SLC16A6
CLEC5A
NLRP3
LOC106781399
F
R: Arabian Mares vs Stallions
Stallion_log2FC
Mare_log2FC
HSPA6
FOSB
FOS
LOC111769297
HSPA1B
NR4A3
RGS1
HSPH1
JUN
DNAJB1
DNAJA1
UGCG
SLC16A6
LOC106781399
